# STXBP6, reciprocally regulated with autophagy, reduces triple negative breast cancer aggressiveness

**DOI:** 10.1002/ctm2.147

**Published:** 2020-08-11

**Authors:** Govinda Lenka, Jingxuan Shan, Najeeb Halabi, Sirin W J Abuaqel, Neha Goswami, Frank Schmidt, Shaza Zaghlool, Atilio Reyes Romero, Murugan Subramanian, Salha Boujassoum, Issam Al‐Bozom, Salah Gehani, Noor Al Khori, Davide Bedognetti, Karsten Suhre, Xiaojing Ma, Alexander Dömling, Arash Rafii, Lotfi Chouchane

**Affiliations:** ^1^ Department of Microbiology and Immunology Weill Cornell Medicine New York USA; ^2^ Genetic Intelligence Laboratory, Weill Cornell Medicine‐Qatar Qatar Foundation Doha Qatar; ^3^ Department of Genetic Medicine Weill Cornell Medicine New York USA; ^4^ Proteomics Core, Weill Cornell Medicine‐Qatar Qatar Foundation Doha Qatar; ^5^ Bioinformatics Core, Weill Cornell Medicine‐Qatar Qatar foundation Doha Qatar; ^6^ Drug Design Group, Department of Pharmacy University of Groningen Groningen Netherlands; ^7^ Department of Medical Oncology National Center for Cancer Care and Research Hamad Medical Corporation Doha Qatar; ^8^ Department of Laboratory Medicine and Pathology Hamad Medical Corporation Doha Qatar; ^9^ Department of Surgery Hamad Medical Corporation Doha Qatar; ^10^ Department of Radiology Sidra Medicine Doha Qatar; ^11^ Tumor Biology section, Research Division Sidra Medicine Doha Qatar

**Keywords:** autophagy, metastasis, STXBP6, triple negative breast cancer, tumor suppressor gene

## Abstract

**Background:**

Although autophagy plays a dual role in suppressing or promoting certain cancers, the nature of its involvement in breast cancers remains unclear. Here, we investigated the function of STXBP6, a protein regulating the autophagy‐associated SNARE complex, in triple negative breast cancer (TNBC).

**Results:**

We report that STXBP6 is profoundly downregulated in TNBC specimens in association with reduced overall patient survival. Notably, we found that *STXBP6* promoter was specifically hyper‐methylated in TNBC specimens. Ectopic expression of *STXBP6* inhibited TNBC cell proliferation in cellular and mouse models. Mass spectrometric analysis revealed physical interactions of STXBP6 with a number of autophagy‐related proteins including SNX27, a molecule involved in endocytosis of plasma membrane receptors and protein trafficking. Overexpression of *STXBP6* elicited autophagy through inhibition of mTORC1 signaling. Reciprocally, induction of autophagy rescued *STXBP6* expression by inhibiting EZH2 and altering *STXBP6* methylation. The mutual regulation between STXBP6 and autophagy was replicated in luminal breast cancer cells only when estrogen receptor (ER) activation was abrogated. Ectopic expression of *STXBP6* significantly reduced TNBC cells’ migratory ability in vitro and tumor metastasis in vivo.

**Conclusions:**

Our results unveil a role of STXBP6 in TNBC that highlights a new paradigm in autophagy regulation. Our results significantly enhance the understanding of the mechanisms of TNBC aggressiveness, which might help in designing novel therapies targeting TNBC.

## 1 BACKGROUND

The biological and pathological diversity of breast cancer results in differences in prognosis and responsiveness to therapy and limited progress in reducing its societal impacts.^1^ The heterogeneous nature of breast cancers is underlined by the variability of mutational pattern resulting in differential expression of genes controlling cell growth and malignant potential.^2^, ^3^ Triple negative breast cancer (TNBC) accounts for approximately 15‐20% of all breast cancers. Despite intensive treatments usually associating surgery, radiation therapy, and chemotherapy, TNBC tumors relapse early and often develop visceral metastases. Hence, TNBC is associated to the poorest prognosis among all breast cancers. TNBC is biologically distinct from other subtypes due to the absence of estrogen receptor, progesterone receptor, and Her‐2 overexpression. Owing to a current lack of targeted therapies (such as hormone therapy or anti‐Her‐2 therapy) for TNBC, non‐selective chemotherapy remains the cornerstone of treatment^4^ and there is an unmet need for novel targeted therapies to improve the outcome for patients with TNBC.

Among the pathways essential to tumor growth, the upregulation of the mTOR pathway plays a pivotal role in TNBC.^5^, ^6^, ^7^, ^8^ Loss of PTEN homolog resulting in activation of the PI3K/PTEN/mTOR pathway, has been reported in 37‐74% of metastatic TNBCs,^9^, ^10^, ^11^ thus making mTOR inhibitors also a potential target for treating TNBC (6‐8). In addition to cell cycle arrest, growth retardation, and reduced angiogenesis, the cellular events mediated by the inhibition of mTOR activity include the activation of autophagy, which is a natural process comprising orderly that is a tightly controlled process comprising lysosomal degradation and recycling of cellular proteins and organelles degradation and recycling of cell components.^12^ Although decreased autophagy appears to be a common hallmark of tumor cells, autophagy has a paradoxical dual role of cytoprotection and cytotoxicity depending on multiple factors including cancer type and stage, tumor microenvironment, and genetic context.^13^, ^14^ On one hand, autophagy can maintain genome integrity, prevent cell injury and inflammation by means of its protein and organelle quality control function as well as by its immune‐surveillance capabilities. In this capacity, it prevents tumor initiation, promotion, and progression, thereby acting as a tumor suppressive mechanism, especially in the early stage of tumorigenesis.^15^, ^16^ On the other hand, autophagy can act as a tumor cells’ protective mechanism and enhances their survival and drug resistance at the late stage of tumor progression.^16^, ^17^ Both oncogenes and tumor suppressor genes regulate autophagy. Commonly mutated oncogenes associated with antiapoptotic proteins and the PI3K/Akt/mTOR pathway possess autophagy inhibitory capacity, so targeting them using rapamycin analogues^18^ and bcl‐2 inhibitors^19^, ^20^ results in the induction of autophagy. Similarly, several tumor suppressive proteins, such as PTEN, tuberous sclerosic complex 1 and 2 (TSC1/2), and the BH3‐only proteins, induce autophagy, while their loss reduces autophagy.^21^ Also, Beclin 1, which is essential for autophagy induction, acts as a haplo‐insufficient tumor suppressor protein.^22^ Thus far, the role of autophagy in breast cancers remains unclear. Unveiling the roles of autophagy throughout different stages of carcinogenesis aids in developing novel therapeutic strategies.

SNAREs have been shown to regulate the initial steps of autophagy including the autophagosome formation.^23^, ^24^ Syntaxin‐binding protein 6 (STXBP6) was identified as a regulator of the autophagy‐associated SNARE complex formation^25^ by binding the syntaxin proteins. Recently, Lenka et al^26^ demonstrated a potential tumor suppressive activity for this protein in lung cancer cells; however, the mechanism underlying antitumor cell growth remains unclear.

The role of STXBP6 in SNARE‐regulation and autophagosome formation led us to hypothesize that constitutive expression of *STXBP6* could influence breast cancer cell behavior through the modulation of autophagy. We started with the assessment of *STXBP6* expression in breast cancer tumor specimen and cell lines. Since we observed a specific methylation‐driven downregulation of *STXBP6* in TNBC, we extended our work with a myriad of biochemical and functional analyses to identify a potential mechanism by which STXBP6 may affect TNBC progression.

## 2 MATERIALS AND METHODS

### 2.1 Clinical tissue samples

Sixty pairs of malignant and adjacent non‐malignant tissues from TNBC patients were collected from the Laboratory Medicine and Pathology and Surgical departments of Hamad Medical Corporation, Doha, Qatar. All participating patients had primary breast cancer, with unilateral tumors and without family history of cancer. The diagnosis of cancer was confirmed by histopathologic analyses. The study was approved by the Weill Cornell Medicine‐Qatar and Hamad Medical Corporation Ethics Committees. All patients gave their written consent for participation in the study.

Tissue specimens were immediately placed in RNAlater (Invitrogen) solution and frozen at −80°C until further use. Frozen tissues were sectioned for the immunohistochemical analysis. DNA and RNA were extracted from tissues using All Prep DNA/RNA/Protein Mini Kit (Qiagen). The quantity and quality of DNA and RNA were measured by NanoDrop™ 2000 (ThermoFisher Scientific).

### 2.2 Cell lines, antibodies, reagents, and virus particles

Breast cancer cell lines (MDA‐MB‐231, MDA‐MB‐468, MCF7, and ZR‐75‐1) and non‐tumoral cell line HEK293T were obtained from American Type Collection Centre (ATCC). Pre‐adipocyte cell line, PAZ6, was kindly gifted by Dr. D. Strosberg.^27^ All above cells were cultured in DMEM/F‐12 medium (GIBCO) supplemented with 10% FBS in a humidified incubator with 5% CO_2_.

Anti‐STXBP6 antibody (HPA003552) was purchased from MilliporeSigma; anti‐SNX27 antibody (ab77799) was purchased from Abcam; others were purchased from Cell Signaling Technology. All chemicals were procured from MilliporeSigma, unless otherwise specified. Lentiviral ORF particles of STXBP6 (RC209598L3V) and SNX27 (RC218477L4V) were purchased from OriGene Technologies. shRNA lentiviral particles targeting STXBP6 (sc‐61968‐V) and SNX27 (sc‐88812‐V) were purchased from Santa Cruz technology.

### 2.3 Quantitative PCR

Total RNA was isolated with TRIzol reagent (Invitrogen) and then was reverse‐transcribed to cDNA with oligo 18T primer. Gene expression were measured by Quantitative PCR (qPCR) using the *hypoxanthine phosphoribosyltransferase 1* (*HPRT1*) gene as a reference. All qPCR assays were performed using SYBR Green‐based GoTaq^@^ 2‐step RT‐PCR System kit (Promega) on a QuantStudio 6 Flex Real‐Time PCR System (Applied Biosystems). The primer sequences are listed in Table S3.

### 2.4 Immunohistochemistry

Formalin‐fixed paraffin‐embedded (FFPE) tissue sections were cut into 5 microns thick and were deparaffinized and rehydrated through three graded concentrations of alcohol. Endogenous peroxidase activity was quenched with 3% H_2_O_2_ for 15 min. Antigen was retrieved in 10 mM pH 6.0 sodium citrate buffer for 20 min at 95°C, followed by 20 min cooling at room temperature. Nonspecific binding was blocked with 5% BSA for 1 h. Sections were incubated with anti‐STXBP6 (1:50) overnight at 4°C and then with HRP‐conjugated secondary antibodies (1:500) for 1 h at room temperature. HRP activity was detected by DAB (DAKO) chromogen. In between steps, sections were washed three times with PBST, each for 7 min. Sections were counterstained with Mayer's hematoxylin and then dehydrated and mounted. Images were taken by a bright field microscope (Carl Zeiss). Section incubated with nonimmune serum acted as a negative control.

### 2.5 Western blot

Cells lysates were prepared in RIPA buffer, diluted in 1× SDS sample buffer and heated for 10 min at 95°C. The total proteins were separated on 10% SDS‐PAGE gel and transferred to a PVDF membrane. Membrane was blocked with 5% milk for 1 h at room temperature and incubated with primary antibody overnight at 4°C, followed by HRP‐conjugated secondary antibody for 1 h at room temperature. In between steps, membrane was washed three times in TBST for 10 min each. The HRP activity was detected by Immobilon® Crescendo (Millipore) and images were acquired by ChemiDOC™ MP (Bio‐Rad).

### 2.6 Immunofluorescence staining

MDA‐MB‐231 cells expressing STXBP6 and its corresponding control cells were grown on culture slides (BD Falcon) to identify STXBP6‐mediated autophagy induction. At 48 h post treatment with 20 nm everolimus, cells were fixed with 4% paraformaldehyde for 15 min at room temperature and permeabilized in ice‐cold methanol for 15 minutes. Cells were then blocked in 5% normal horse serum for 1 h at room temperature and probed with rabbit anti‐LC3 and mouse anti‐STXBP6 antibodies overnight at 4°C. Cells were incubated with anti‐mouse IgG‐AlexaFluor‐488 (green) and anti‐rabbit IgG‐AlexaFluor‐594 (red) (1:200) secondary antibodies for 1 h at room temperature and then loaded with DAPI for nuclear staining. The coverslips with the attached cells were finally mounted on glass slides with fluorescent mounting media (Vector Laboratories). The fluorescence images were captured using a LSM710 confocal laser scanning microscope (Carl Zeiss).

### 2.7 Pyrosequencing assay

Bisulfite treatment of genomic DNA was carried out using an EpiTect Fast bisulfite conversion kit (Qiagen) following the manufacturer's instructions. Then a predesigned PyroMark CpG Assay (PM00057414, Qiagen) was used for quantification of CpG methylation of *STXBP6* gene according to the manufacturer's instruction.

### 2.8 Methylation‐specific PCR

Total cellular DNA from autophagy induced TNBC cells and their corresponding controls were isolated and purified using DNeasy blood and tissue kit (Qiagen). The DNA was then subjected to bisulfite conversion using an EpiTect Fast bisulfite conversion kit with a program of 16 cycles for 30 s at 95°C and 1 h at 50°C. The converted DNA was subjected to a methylation‐specific PCR reaction using both unmethylated primer pairs (For (U): TGAGTATGTTTAGAGGTGGTT and Rev (U): AACTTAACCAACCCAAATAC) and methylated primer pairs (For (M): GAGTATGTTTAGAGGCGGTC and Rev(M): ACTTAACCGACCCGAATAC) (36).

### 2.9 Cell proliferation assay

Cells in 96‐well plated were incubated with growth medium containing 1 mg/mL MTT for 3 h at 37°C followed by incubation with 100 μL dimethyl sulfoxide agitated on an orbital shaker for 15 min covered with tinfoil. Absorbance was recorded at 570 nm with CLARIOstar (BMG Labtech).

### 2.10 Colony formation assay

One thousand cells were seeded in a well of six‐well plates and cultured for 2 weeks to form colonies. Then cells were fixed with methanol:acetic acid (3:1) and stained with 0.1% crystal violet. Finally, the images of dried plates were acquired with a digital camera.

### 2.11 Cell migration assay

Cells were seeded and cultured to create a confluent monolayer in six‐well plate. The cell monolayer was scratched with a 200 μL pipet to produce a line, washed one time with growth medium to remove the debris and then incubated in growth medium to acquire the first image of the scratch using a phase‐contrast microscope. The cells were cultured in normal condition and the scratch was imaged every 24 h.

### 2.12 Co‐immunoprecipitation

Cells were lysed using immunoprecipitation (IP) lysis buffer (ThermoFisher Scientific) and centrifuged at 12 000 × *g* for 15 min at 4°C. For each IP, cell lysates containing 500 μg total protein were first precleared with 20 μL agarose slurry for 3 h at 4°C on a rotator. Precleared lysates were mixed with 5 μg anti‐myc for 2 h at room temperature and then incubated with pre‐blocked agarose slurry overnight at 4°C on a rotator. Beads with IP product were washed five times with PBST and three times with PBS, resuspended in 40 μL 2x Laemmli Sample Buffer (ThermoFisher Scientific) and boiled for 10 min. The supernatant was subjected to western blot analysis.

### 2.13 Proteome analysis using gel‐LC‐MS/MS

IP samples were subjected to proteomics analysis according to in‐gel digestion method of Rosenfeld et al.^28^ The IP samples were run in a 1D‐gel (NuPage 4‐12% Bis‐Tris gel, 10‐well 1.0 mm) for approximately 10 min until a clear single band was visible. The band of each sample was then carefully excised and cut into smaller pieces between 1 and 2 mm^2^. Gel were transferred to clean tubes and covered with dithiothreitol and iodoacetamide buffers for reduction and alkylation of the proteins, respectively. The gel pieces were then covered with sufficient trypsin solution for overnight digestion into peptides at 37°C. The peptides were extracted from the gel pieces using various concentrations of acetonitrile and ethanol mixed with ammonium bicarbonate buffer and dried down in a speedvac. The peptides were then purified using Purespeed tips, dried down in the speedvac, and acidified using 0.5% formic acid to run in the Q‐Exactive HF (Thermo Fisher Scientific). The mixed peptides were then subjected to nano‐liquid chromatography (LC) coupled with mass spectrometry; the analytical platform consisting of an EASY nLC‐1200 interfaced to a Q‐Exactive HF. Chromatography conditions were defined as follows: A, water with 0.5% acetic acid ; B, water:acetonitrile (20:80) with 0.5% acetic acid; C, 250 nL/min flow rate, 6.0 μL injection volume, and a maximal loading pressure of 280 bars. LC‐separation was run on 18 cm long in‐house packed emitter columns (ReproSil‐Pur 120 C18‐AQ 3 μm diameter beads, kindly provided by Dr. Maisch GmbH). For the first trial, samples were run using a gradient ranging from 2% to 95% mobile phase B over 160 min, followed by a 10‐min wash and column re‐equilibration cycle. Data dependent mass spectrometric acquisition employed a top 20 method. Briefly, the 20 most intense ions, excluding unassigned‐charge states and singly charged ions, detected in the preceding full scan were isolated (1.2 Th isolation width) and fragmented using higher energy collisional dissociation (HCD) (normalized collision energy 28). Precursor scans were acquired at a resolution of 60 000 (scan range of m/z 400‐1650) and an AGC target value of 3 000 000 charges (maximum ion injection time 20 ms). MS/MS fragmentation spectra were acquired at a resolution of 15 000 and an AGC target value of 100 000 charges (maximum ion injection time 120 ms) with a fixed lower mass‐to‐charge cut‐off of 100. All scan events were recorded in profile mode, a dynamic exclusion list of 90s was employed, and both exclude isotopes and peptide match functionalities were activated.

### 2.14 MS data analysis

Raw MS data were processed using Genedata Expressionist software (v.13.0.1), consisting of two modules: Refiner MS (data preprocessing) and Analyst (data post‐processing and statistical analysis). Briefly, after noise reduction and normalization, LC‐MS peaks were detected and their properties calculated (*m*/*z* and RT boundaries, *m*/*z* and RT center values, intensity). Individual peaks were grouped into clusters and MS/MS data associated with these clusters were annotated with MS/MS Ions Search (Mascot 2.6.2) using peptide tolerance: 10.0 ppm; MS/MS tolerance: 0.50 Da; maximum missed cleavages: 2; and database: Uniprot Swiss‐Prot 29062016, taxonomy *Homo sapiens*. Results were validated by applying a threshold of 1% corrected normalized false discovery rate (FDR). Due to the low complexity, peptides with individual ions scores >31 only (indicating identity or extensive homology, *P *< .05) were also taken into account. Protein interference was done based on peptide and protein annotations. Redundant proteins were ignored according to the Occam's razor principle, and at least one peptide was required for a positive protein identification. Protein intensity ranks (for Table S1) were computed using the Hi3 method.^29^

### 2.15 Docking analysis

Homology models of the N‐terminal Sec3‐PIP2 domain from human STXBP6 and PDZ domain from Sorting Nexin‐27 (SNX27) were prepared and evaluated with SWISS‐MODEL,^30^ using 3HLE and 4Z8J, respectively, as templates, while the crystal structure of 78‐kDa glucose‐regulated protein (GPR78) was fetched from the Protein Data Bank (PDB) under the accession number 3IUC. Subsequently, the proteins coordinates were docked using ClusPro 2.0,^31^ implementing the mass spectrometry peptide sequences of SNX27 and GPR78 as attraction restrains. Top best poses corresponding to highly populated clusters from the four different contributions of the docking algorithm (balanced, electrostatic, hydrophobic, and van der Waals + electrostatic) were downloaded, and the solvation‐free energy gain (ΔiG) and interface area were calculated with PISA.^32^, ^33^ Finally, the intermolecular interactions were analyzed with Scorpion^34^ and the figures were rendered with Pymol (The PyMOL Molecular Graphics System, Version 2.0 Schrödinger, LLC). The primary sequences used for homology modeling are as follows:

SEC3‐PIP2 from STXBP6
QGEYLTYICLSVTNKKPTQASITKVKQFEGSTSFVRRSQWMLEQLRQVNGIDPNGDSAEFDLLFENAFDQWVASTASEKCTFFQILHHTCQRY
PDZ domain
GPRVVRIVKSESGYGFNVRGQVSEGGQLRSINGELYAPLQHVSAVLPGGAADRAGVRKGDRILEVNHVNVEGATHKQVVDLIRAGEKELILTVLSV

### 2.16 Xenograft mouse models

Seven to 8 weeks old female severe combined immunodeficiency (SCID) mice, weighting from 17 to 19 g, were obtained from Shanghai SLAC Laboratory Animal Co. Ltd, and housed in the PharmaLegacy Laboratories vivarium. The adaptation to the environment for the mice was no less than 7 days. Twelve mice were randomly assigned to two groups for each experiment according to their body weight. The procedures that were applied in this study have been approved by PharmaLegacy Laboratories Institutional Animal Care and Use Committee. All the included mice were euthanized at their scheduled study termination.

For the orthotopic models, 5 × 10^6^ STXBP6‐overexpressing MDA‐MB‐2 cells, *STXBP6* knockdown MDA‐MB‐231 cells or their corresponding mock cells in PBS were injected into the mammary fat pad of each mouse, and the tumor volume and mouse body weight were measured every 4 days once palpable tumors appeared. After 4 weeks, to evaluate the effect of STXBP6‐overexpression on tumor growth, the tumors were collected; to evaluate effect of autophagy activity on xenograft tumor growth, 1 mg/kg everolimus in PBS was injected intratumorally at 4‐day intervals for another 3 weeks. Finally, the xenograft tumors were excised, weight and examined by IHC.

For metastasis models, approximately 1 × 10^5^ luciferase‐labeled control (mock) and *STXBP6‐Lenti* MDA‐MB‐231 cells suspended in PBS were injected via intracardiac route^35^ to each mouse under anesthesia by 3‐4% isoflurane. The distribution and mean fluorescence index (MFI) of the tumor were measured by the IVIS in vivo imaging system (PerkinElmer) once a week for 7 weeks.

### 2.17 TCGA expression analysis

We downloaded the TCGA Breast Cancer RNA Sequencing Expression Data (https://portal.gdc.cancer.gov) using the GDC DTT Desktop tool on February 29, 2020. We identified the count data files (htseq.count files) and then filtered and scaled the count data using TMM normalization with edgeR 3.28.1.^36^ The filtered and scaled expression dataset contained 18 514 genes. The expression dataset was then further analyzed in Matlab with custom scripts. To identify the TNBC subset in the TCGA dataset, we used the TNBC samples described in Jiang et al^37^ that resulted in identifying 121 TNBC samples containing 113 cancer samples and eight normal solid tissue. We calculated the gene expression profiles of autophagy genes in TNBC and nonTNBC samples using the Matlab 2019a boxplot function using the default settings. We calculated the Kendall Tau rank correlation between *STXBP6* expression and other autophagy gene expression using the corr function in Matlab 2019a with default settings.

### 2.18 Statistical analysis

Data were expressed as the means ± SDs from at least biological triplicates. The statistical significance in different samples was analyzed by a *t*‐test. Time course difference between two groups were analyzed using two‐way ANOVA. All statistical tests were two‐sided. *P*‐values > .05 were considered significant. Statistical analyses were performed using GraphPad Prism 7.

## 3 RESULTS

### 3.1 Correlation of overall breast cancer patient survival with *STXBP6* expression

**FIGURE 1:**
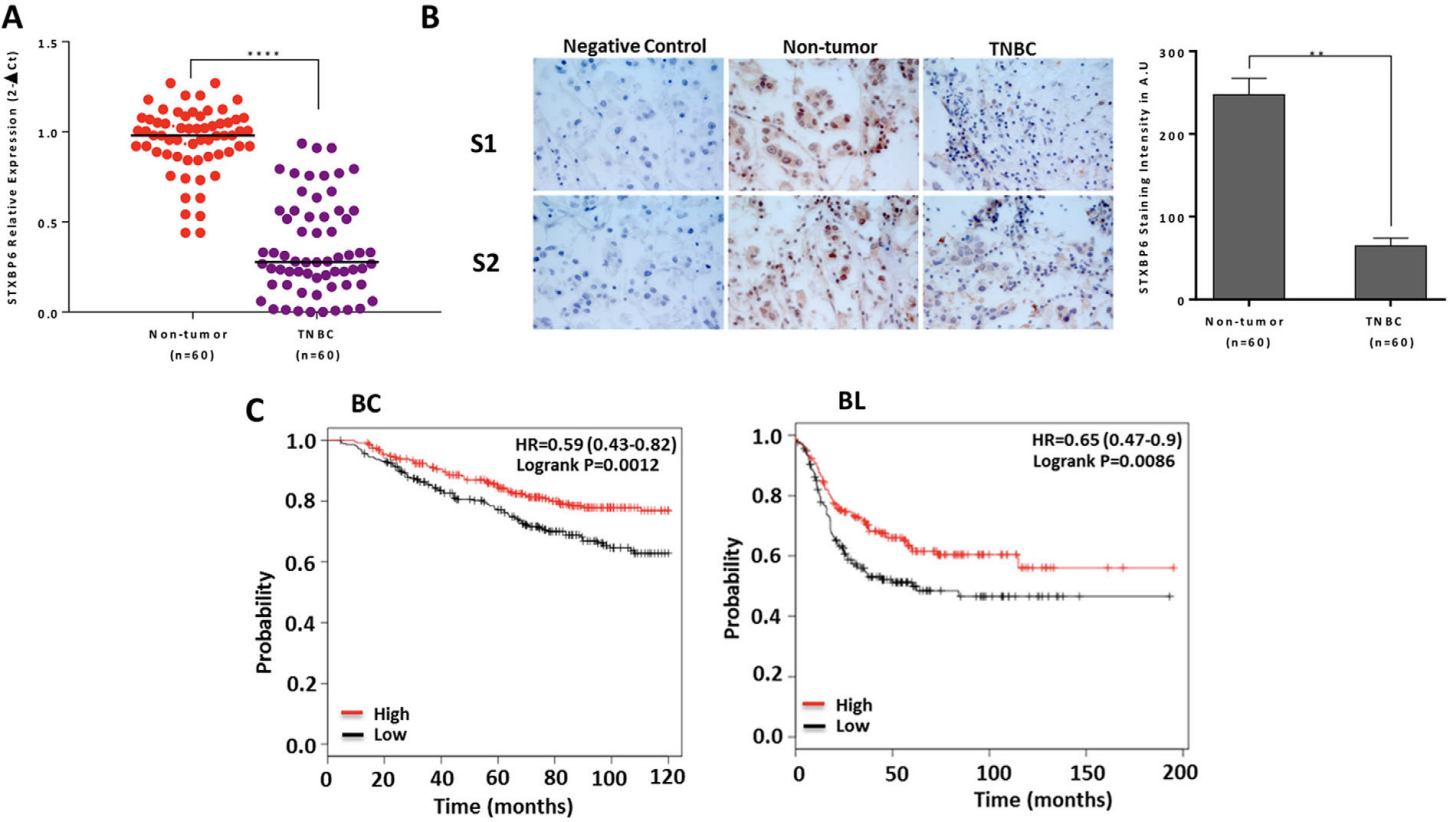
Clinical analysis of STXBP6 in breast cancer. A, *STXBP6* mRNA levels in 60 triple negative breast cancer (TNBC) tumor specimens and their corresponding nonmalignant breast tissues. Relative *STXBP6* expression was calculated by 2^−ΔCt^. The expression differences between two groups were analyzed by a t‐test. ****, *P *< 0.0001. B, The representative images of immunohistochemical staining of STXBP6 in two TNBC specimens (S1 and S2) and their corresponding non‐malignant breast tissues (magnification, ×40). Non‐malignant breast tissues were also used as negative control in which the tissues were incubated without primary antibody (left). The mean values of the IHC quantification in arbitrary units (A.U.) are shown in right. ^**^ *P *< .01 indicate the significant difference in STXBP6 immunoreactivity between tumor and nontumor breast tissues, which was analyzed by a *t*‐test. n = 60, The number of patients included in the study. Error bars represent the standard deviation of 60 replicates. C, Overall survival according to the *STXBP6* expression levels (high vs low, using the median as cutoff) in all types of breast cancer patients (BC) and basal‐like breast cancer patients (BL) of Kaplan‐Meier plotter dataset. The differences between groups were calculated by the log‐rank test. “|” represents each censored sample

We first examined the expression of *STXBP6* at mRNA and protein levels in 60 pairs of TNBC tissues and their corresponding nontumor breast tissues. We observed significant downregulation of *STXBP6* mRNA in the tumor tissues relative to its adjacent nontumor breast tissues (*P *< .0001; Figure 1A). We further showed the downregulation of *STXBP6* expression at a protein level by immunohistochemistry (IHC) in the same tumor tissues; STXBP6‐staining intensity in tumors compared with nontumor samples was found to be significant (*P *< .01; Figure 1B). Next, we analyzed breast cancer patient survival using a Kaplan‐Meier plotter (www.kmplot.com/breast, ref. 38). As shown in Figure 1C, high expression of *STXBP6* was associated with an increased overall survival (OS) in all breast cancer patients, particularly in TNBC/basal‐like breast cancer patients. We also analyzed patient survival using TCGA datasets.^39^ Similarly, high expression of *STXBP6* was associated with an increased overall survival in TCGA BC cohort (Figure S1). Taken together, these findings suggest a role for STXBP6 in TNBC biology and the potential prognostic value of STXBP6 to predict TNBC disease progression.

### 3.2 Control of *STXBP6* expression via promoter methylation in TNBC cells

**FIGURE 2:**
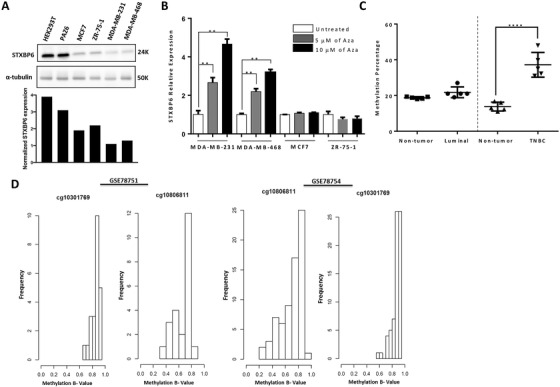
Methylation analysis of *STXBP6* in breast cancer. A, Upper: western blotting of endogenous STXBP6 using anti‐STXBP6 antibodies in luminal (MCF7 and ZR‐75‐1) and triple negative (MDA‐MB‐231 and MDA‐MB‐468) breast cancer cells and non‐malignant control cells (HEK‐293T and the brown pre‐adipocyte cell line PAZ6). Equal loading was assessed by α‐tubulin. Lower: normalized expression of STXBP6 relative to α‐tubulin in the indicated cells. The band intensity was measured by ImageJ. B, Relative expression of *STXBP6* mRNA in breast cancer cells treated with 5 and 10 μM of methylation inhibitor 5‐aza‐2‐deoxycytidine (5‐Aza) for 3 days. Relative expression levels of *STXBP6* were normalized against the untreated group of each cell line. The expression difference was analyzed by *t*‐test. ^**^ *P *< .01. Error bars represent the *SD* of biological triplicates. C, Quantification of DNA methylation of *STXBP6* in breast cancer tissues and their adjacent nonmalignant breast tissues. Each subtype of breast cancer tissue samples was compared to the corresponding nonmalignant tissue samples. The methylation percentage difference was analyzed by *t*‐test. ^****^ *P *< .0001. D, Determination of methylation status of *STXBP6* in TNBC samples of public datasets (GSE78751 and GSE78754). Histograms of the 2 CpG sites (cg10301769 and cg10806811) in the 5‘‐UTR of *STXBP6* gene. β‐Value > 0.4 indicates hypermethylation

To determine the mechanisms underlying the repression of *STXBP6* expression in breast cancer cells, we first examined the endogenous expression of *STXBP6* in TNBC subtype (MDA‐MB‐231 and MDA‐MB‐468), Luminal subtype (MCF7 and ZR‐75‐1), and non‐malignant cells (HEK293T and pre‐adipocyte cell line, PAZ6). In agreement with the clinical specimen results, STXBP6 was downregulated in all breast cancer cells included in this study (Figure 2A). Notably, the STXBP6 in TNBC cells is repressed more than in luminal breast cancer cells. In cancer, the downregulation of gene expression often ascribes to its promoter methylation. H3K27ac mark represents a hot spot where DNA methylation often occurs.^40^ Accordingly, strong H3K27ac enrichment was found on the *STXBP6* promoter region based on UCSC genome browser data (Figure S2), indicating that *STXBP6* expression might be silenced due to promoter region methylation in cancer cells. We verified the effect of methylation on *STXBP6* expression by treating breast cancer cells with the DNA methyltransferase (DNMT) inhibitor, 5‐aza‐2′‐deoxycytidine (5‐Aza). *STXBP6* expression was significantly upregulated only in TNBC cells in a dose‐dependent manner (*P *< .01), but not in luminal subtype breast cancer cells (Figure 2B). Further, pyrosequencing analysis revealed the *STXBP6*‐specific CpG island methylation. A total of five CpG sites including Chr14: 25518720, 25518735, 25518737, 25518743, and 25518748 in the 5′ untranslated region (UTR) of *STXBP6* were examined. This analysis revealed the higher methylation percentage of four of the five CpG sites in all TNBC tissues compared to luminal breast cancer type (*P *< .01) and to nontumor breast tissue samples (*P *< .0001) included in this study (Figure 2C; Figure S3). In addition, in silico methylation analysis using public data sets showed also that the promoter of *STXBP6* was hypermethylated in TNBC (Figure 2D). Together, all the above results strongly indicate that epigenetic control (methylation) of *STXBP6* expression might be specific to TNBC cells.

### 3.3 Inhibition of tumor cell proliferation by STXBP6

**FIGURE 3:**
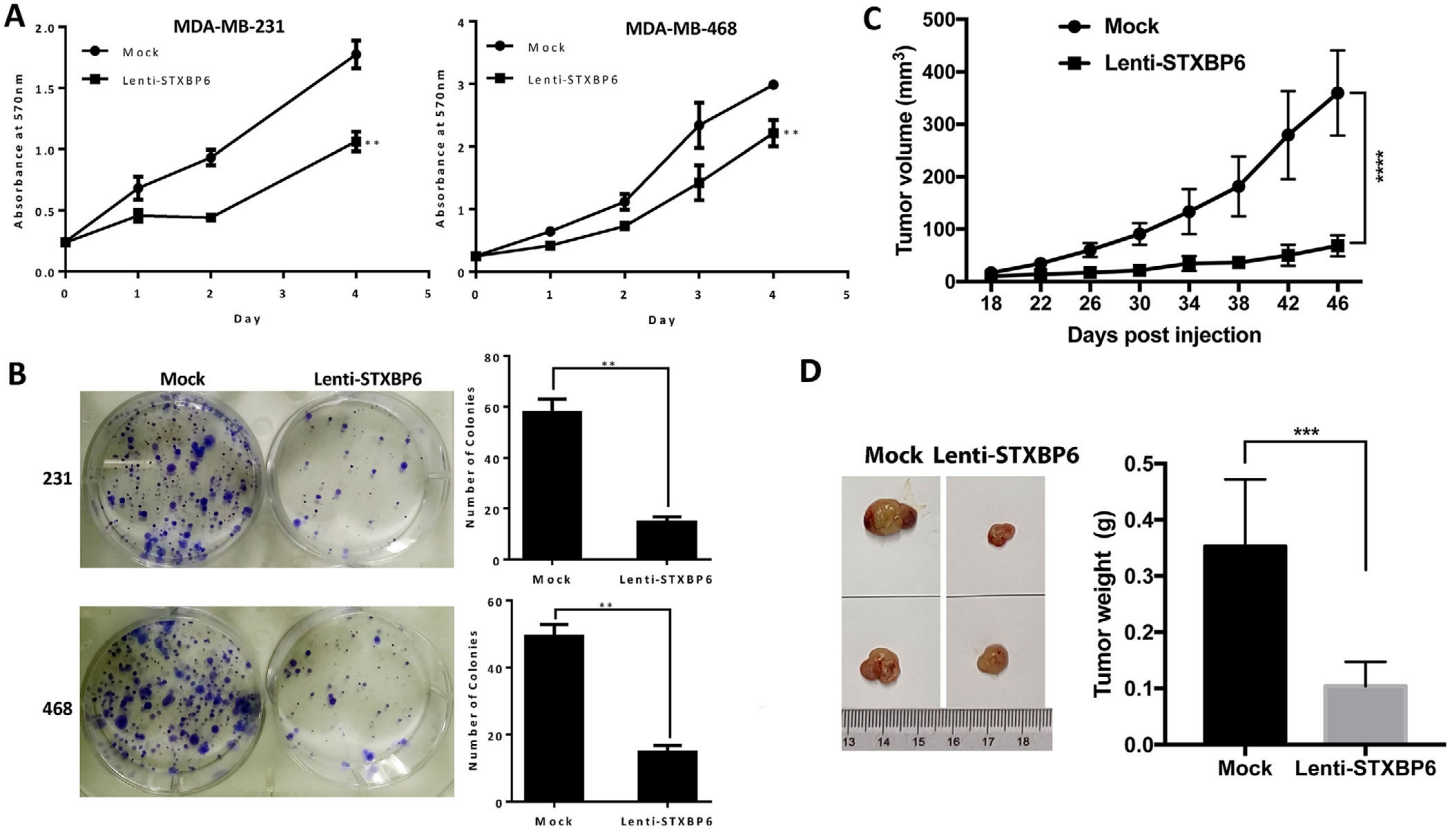
The effect of *STXBP6* overexpression on breast cancer cell growth in vitro and in vivo. A, Proliferation assays of TNBC cells overexpressing *STXBP6* (Lenti‐STXPB6) versus empty vector (Mock). Cells at a concentration of 5000 cells per well were seeded in the 96‐well plate and incubated for indicated time. The quantity of viable cells was determined by MTT assay. The growth difference between Lenti‐STXPB6 and mock groups was analyzed by two‐way ANOVA. ^**^ *P *< .01. B, Colony formation assays of MDA‐MB‐231(left upper panel) and MDA‐MB‐468 (left down panel) TNBC cells overexpressing *STXBP6* (Lenti‐STXPB6) compared with the control (Mock). Left, The whole‐well images of clonogenic cells; right panels, the average number of colonies ± *SD*. The difference of colony formation ability between Lenti‐STXPB6 and mock groups was analyzed by *t*‐test. ^**^ *P *< .01. C, Orthotopic tumor growth curve of STXBP6‐overexpressing MDA‐MB‐231 cells (Lenti‐STXPB6) and control (Mock) MDA‐MB‐231 cells after palpable tumor appeared. The tumor growth between Lenti‐STXPB6 and mock groups was compared by a two‐way ANOVA. ^****^ *P *< .0001. D, Left: representative images of tumor size. Right: average tumor weight of each group. The tumor size between Lenti‐STXPB6 and mock groups was compared by a *t*‐test. ^***^ *P *< .001. Error bars represent the standard deviation of biological triplicates (A, B) or six replicates (C, D)

The clinical data analysis of STXBP6 indicated an antitumor role of STXBP6. To investigate the role of STXBP6 in TNBC, we developed overexpressing *STXBP6* (Lenti‐STXPB6) TNBC cells by infecting MDA‐MB‐231 and MDA‐MB‐468 cells with a lentiviral system carrying the *STXBP6* gene. We validated *STXBP6* overexpression at both mRNA and protein levels (Figure S4). Using MTT assay, we showed that *STXBP6* overexpression caused a significant reduction in proliferation of both TNBC cells compared to controls (*P* ≤ .01; Figure 3A), and shRNA‐mediated knockdown of *STXBP6* had no significant effect on cell proliferation (Figure S5A). We then performed a clonogenic assay, which showed that *STXBP6* overexpression significantly decreased the rate of colony formation (Figure 3B). Conversely, knockdown of *STXBP6* promoted colony formation in both MDA‐MB‐231 and MDA‐MB‐468 cells (Figure S5B). To further confirm that STXBP6 inhibits TNBC cell growth, Lenti‐STXBP6 cells and mock cells were tested in a *xenograft* mouse model. We observed that tumors in the Lenti‐STXBP6 group were significantly reduced (5‐ to 6‐fold) compared to the control group (Figure 3C,D).

### 3.4 Identification of STXBP6‐interacting proteins in TNBC cells

**FIGURE 4:**
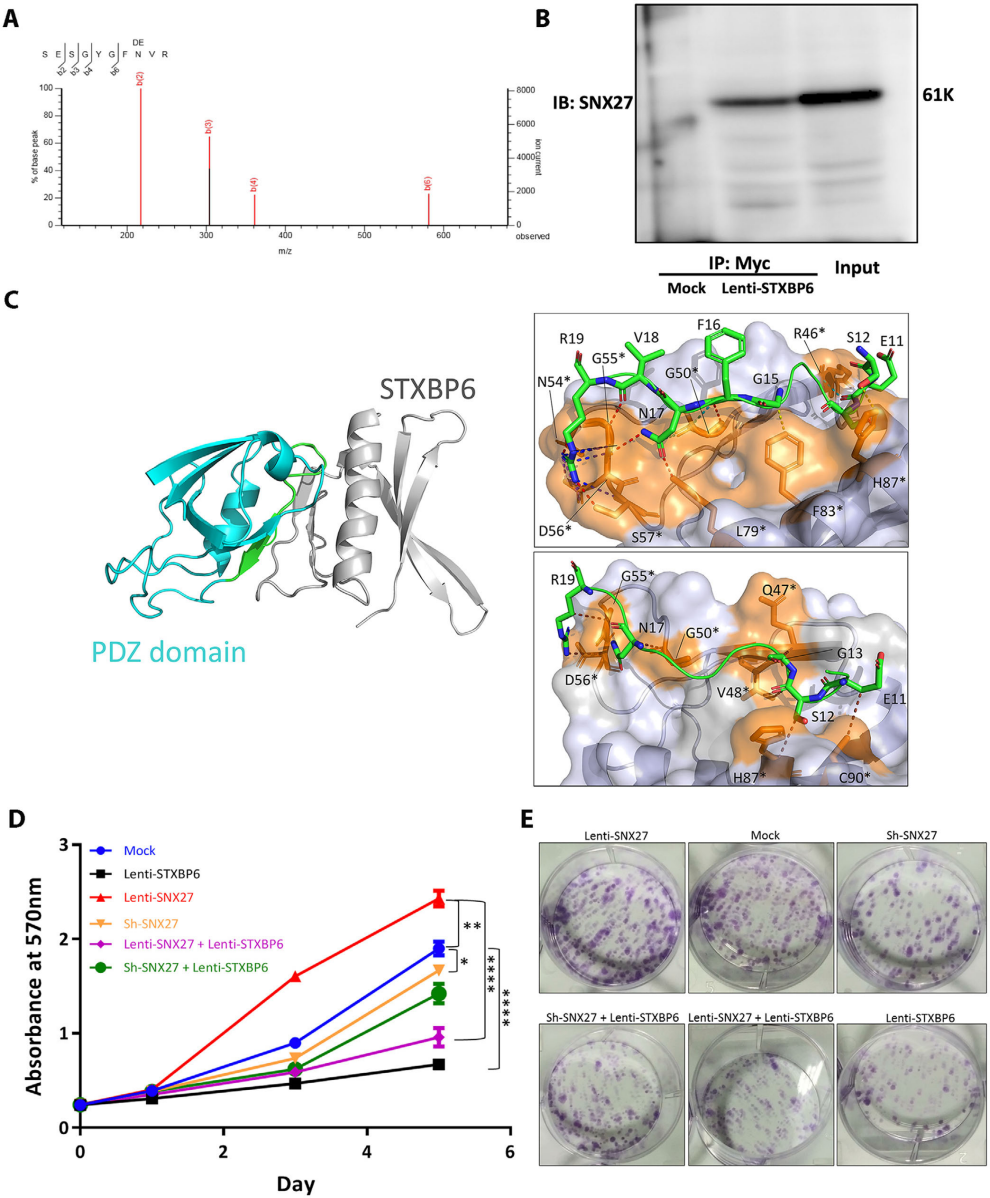
STXBP6 interacts with SNX27. A, Data‐dependent MS/MS fragment spectra of peptide identifying SNX27. B, Co‐IP of STXBP6 and SNX27. Proteins immunoprecipitated from total cell lysates of MDA‐MB‐231 cells overexpressing *STXBP6* (Lenti‐STXPB6) versus empty vector (Mock) using an anti‐Myc antibody. Bands of 61K corresponding to SNX27 were identified in the IP product from cells overexpressing *STXBP6* or in total lysate of cells but not in the IP product of mock cells. C, Cartoon representation of the docking model of STXBP6 (grey cartoon) in complex with PDZ domain of SNX27 (cyan cartoon). Amino acidic sequence used for restraining mode in ClusPro 2.0 is colored in green. Protein interactions of STXBP6 (green sticks) and PDZ domain of SNX27 (orange sticks and grey surface) generated with Scorpion consist of a complex network of van Der Walls (brown dotted lines Right upper); Right lower: hydrogen bonds, cation‐π stacking, dipolar, ionic contacts (red, blue, cyan, and violet dotted lines), van der Waals interactions (brown dotted lines). ^*^Indicates molecular contacts. D, Proliferation assays of engineered MDA‐MB‐231 cells as indicated. The cell proliferation between any two groups was compared by a two‐way ANOVA. ^*^ *P *< .05; ^**^ *P *< .01; ^****^ *P *< .0001. Error bars represent the standard deviation of biological triplicates. E, Colony formation assays of engineered MDA‐MB‐231 cells as indicated

To unveil the molecular mechanism by which STXBP6 inhibits tumor cell growth, we identified the interacting partners of STXBP6. We immunoprecipitated all STXBP6‐interacting proteins in Lenti‐STXBP6 MDA‐MB‐231 cells and control cells. Immunoprecipitated proteins were gel excised, protein digested with trypsin, and analyzed by nano‐LC coupled to mass spectrometry (MS). A complete list of identified binding partners of STXBP6 is provided in Table S1, and the MS/MS spectra for peptides matching to STXBP6 are shown in Figure S6. Given its biological function in breast cancer, we validated the interaction of SNX27 with STXBP6 by co‐immunoprecipitation (Figure 4A). Furthermore, we performed docking of this interaction. The proposed docking models of STXBP6 in complex with the PDZ domain of SNX27 are shown in Figure 4B, Left. The interface formed between STXBP6 and the PDZ domain of SNX27 (950 Å2) accounts for 14% of the total complex with a solvation free energy ΔiG of −3.5 kcal/mol. The interface is mainly composed of hydrophobic interactions and additionally, we counted 11 hydrogen bonds and eight salt bridges. Three out of four of the best docking poses effectively matched the SNX27‐PDZ domain epitope. Scorpion analysis revealed the presence of seven hydrogens bond from several amino acids of PDZ domain, for instance between the R19 guanidine side chain and N54, D56, and S57 of STXBP6. Similarly, further contacts were observed from the CO moieties of V18 and F16 with G55 and G50. Moreover, other hydrogen bonds are formed by N17 side chain amide group with both L79 and G55 and finally between S12 side OH moiety and H87 (Figure 4B, right upper). Additional stabilizing interaction consisted of cation‐π stacking and ionic contacts between guanidine R18 and G55, S57, and D56 of STXBP6. N17 and F16 from the PDZ domain provided further dipolar contacts with G50 backbone carbonyl of PDZ domain, and, finally, seven van der Waals interactions from R19, N17, G13, S12, and E11 (Figure 4B, right lower). Interestingly, highest Scorpion scores were found on the R19 side chain of SNX27‐PDZ domain through four ionic contacts and one cation‐π stacking, and additionally on the R53 guanidine group, which interacts with the CO‐moiety of Q91 of STXBP6 through two confocal hydrogen bonds.

The oncogenic role of SNX27 in TNBC has been well established,^41^ which is diametrically opposite to STXBP6. The expression of *SNX27* is upregulated in patients with invasive breast cancer. Depletion of SNX27 inhibited proliferation and metastasis of TNBC cells in vitro and in vivo.^42^ Both STXBP6 and SNX27 are implicated in the process of membrane fusion. Thus, we hypothesize that STXBP6 binds to SNX27 and then suppresses its oncogenic function. As shown in Figure 4C,D, *STXBP6* overexpression significantly inhibited TNBC‐cell proliferation and colony formation ability when *SNX27* is expressed or overexpressed, but the degree of STXBP6‐induced inhibition was significantly reduced when *SNX27* is depleted.

### 3.5 Promotion of autophagy by STXBP6 through the mTOR pathway

**FIGURE 5:**
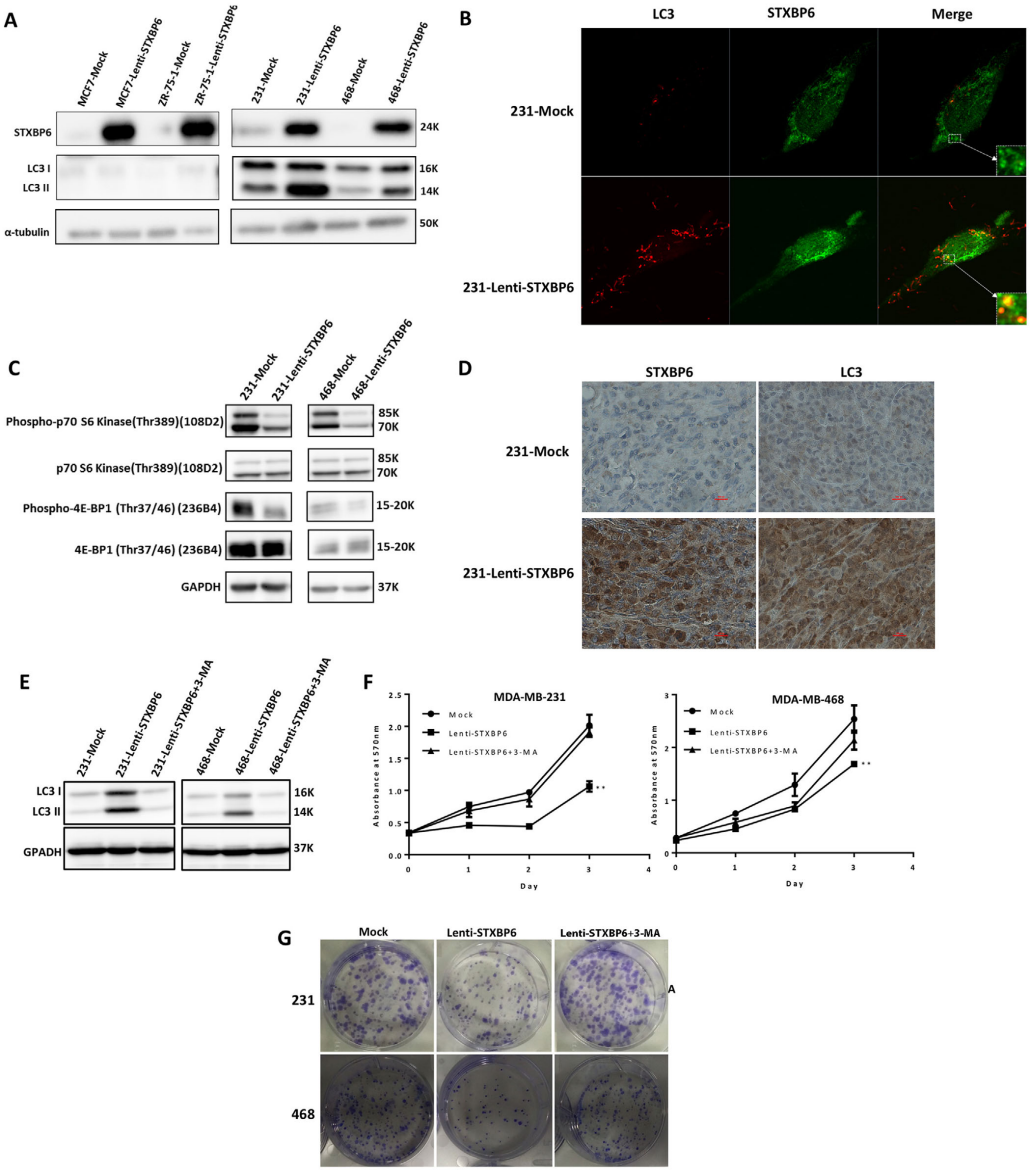
STXBP6 triggers autophagy in TNBC cells by inhibiting mTOR pathway. A, Western blot analysis using LC3 antibodies in STXBP6‐overexpressing (Lenti‐STXPB6) and control (Mock) TNBC cells and in luminal subtype breast cancer cells. Equal loading was assessed by α‐tubulin. B, Immunofluorescence analysis of endogenous LC3 and STXBP6 in mock and STXBP6‐overexpressing MDA‐MB‐231 cells. Cells were stained with mouse anti‐LC3 and rabbit anti‐ STXBP6 antibodies, followed by anti‐mouse IgG‐AlexaFluor‐594 (red) and anti‐rabbit IgG‐AlexaFluor‐488 (green) secondary antibodies. Representative images are shown at 40× magnification. Note that LC3 (red) is clearly seen in STXBP6 stained in Lenti‐STXPB6 cells (green). C, Western blotting analysis of the activity of mTOR downstream associated molecules Phospho‐p70 S6 Kinase, p70 S6 Kinase, Phospho‐4E‐BP1, and 4E‐BP1 in Lenti‐STXPB6 and control cells. Equal loading was assessed by GAPDH. D, Immunohistochemical analysis of LC3 and STXBP6 in TNBC xenograft tumors from mice inoculated with STXBP6‐overexpressing MDA‐MB‐231 cells and control group (inoculated with the corresponding Mock cells). Magnification: 400×. E, STXBP6‐overexpressing cells were treated with autophagy inhibitor, 3‐methyl adenine (3‐MA). Western blotting analysis of LC3 suggested that the induced autophagy in STXBP6‐overexpressing cells was repressed by 3‐MA. F, Inhibition of autophagy removed the repression of STXBP6 on tumor cell growth. Cell growth was determined by MTT assay. The difference between two groups was analyzed by a two‐way ANOVA. ^**^ *P *< .01. Error bars represent the standard deviation of biological triplicates. F, Colony formation assays of STXBP6‐overexpressing cells with or without the treatment of 3‐MA compared to the control (Mock)

The activity of STXBP6 in regulating autophagy‐associated SNARE complex formation together with the demonstrated role of SNX27 in activating the mTOR pathway and inhibiting autophagy^41^ suggest that STXBP6 may regulate autophagy. Furthermore, MS data showed that autophagy‐related proteins such as SNX27, GRP75, GRP78, ABCA13, and SNAP29 were enriched in STXBP6‐interacting proteins (Figure S6). To get insights into the role of STXBP6 in autophagy, we assessed the autophagy markers in Overexpressing *STXBP6* (Lenti‐STXPB6) TNBC cells. As shown in Figure 5A, right panel, Western blot analysis showed a markedly increased expression of the autophagy LC3‐II marker in both of the Lenti‐STXBP6 TNBC cells. We also observed an enhancement of endogenous LC3 in punctate structures (Figure 5B). Interestingly, STXBP6‐induced autophagy was not detected in luminal‐subtype breast cancer cells (Figure 5A, left panel). Furthermore, we selected conserved and important autophagy pathway genes (Table S2) from the Autophagy Database (http://www.tanpaku.org/autophagy/index.html, ref. 43) and analyzed the expression profiles of STXBP6 and these genes in the TCGA breast cancer cohort. We found that *STXBP6* was among the autophagy genes with the lowest expression in TNBC (Figure S7) and that over 70% of autophagy pathway genes vary with *STXBP6* (*P* < .05) in TNBC (Figure S8), indicating that the autophagy pathway genes are frequently downregulated in TNBC and *STXBP6* might serve as a marker to evaluate whether autophagy pathway is induced or inhibited. To determine whether *STXBP6* expression is correlated with specific genes in TNBC, we compared the rank of the *P*‐value of the Kendall correlation in TNBC and non‐TNBC of the TCGA dataset, and found that *STXBP6* correlated with several genes, including *BCL2, NUAK2, CDK2, ATG13, CAMK1, MTOR, POC1B*, and *ZFYVE1*, only in TNBC (Figure S9). We quantified the expression of several autophagy genes, including *BECN1, ATG9B, SQSTM1*, in Lenti‐STXBP6 TNBC and luminal type cell lines, and the results were consistent with TCGA data (Figure S10). These results demonstrated that *STXBP6* overexpression triggered autophagy only in TNBC cells.

The mTOR is a negative regulator of autophagy and the activity of mTOR pathway determine autophagy activity.^44^ We examined the phosphorylation of mTOR substrate proteins including p70 S6 kinase and 4E‐BP1 and both were inhibited in Lenti‐STXBP6 TNBC cells (Figure 5C), suggesting that STXBP6 induced autophagy by repressing the mTOR pathway. We further confirmed the activation of autophagy by STXBP6 in the TNBC mouse model. As shown in Figure 5D, IHC analysis of tumors from mice inoculated with Lenti‐STXBP6 TNBC cells shows high expression of both STXBP6 and LC3 autophagy marker. We next investigated the functional significance of autophagy for the tumor suppressive activity of STXBP6. An autophagy inhibitor, 3‐methyl adenine (3‐MA), inhibited the STXBP6‐induced autophagy (Figure 5E) and restored the proliferation ability (Figure 5F) and the colony formation ability (Figure 5G) of Lenti‐STXBP6 TNBC cells, suggesting an indispensable role of autophagy in STXBP6 mediated tumor suppression.

### 3.6 Autophagy induction‐mediated rescue of STXBP6 expression by inhibiting EZH2

**FIGURE 6:**
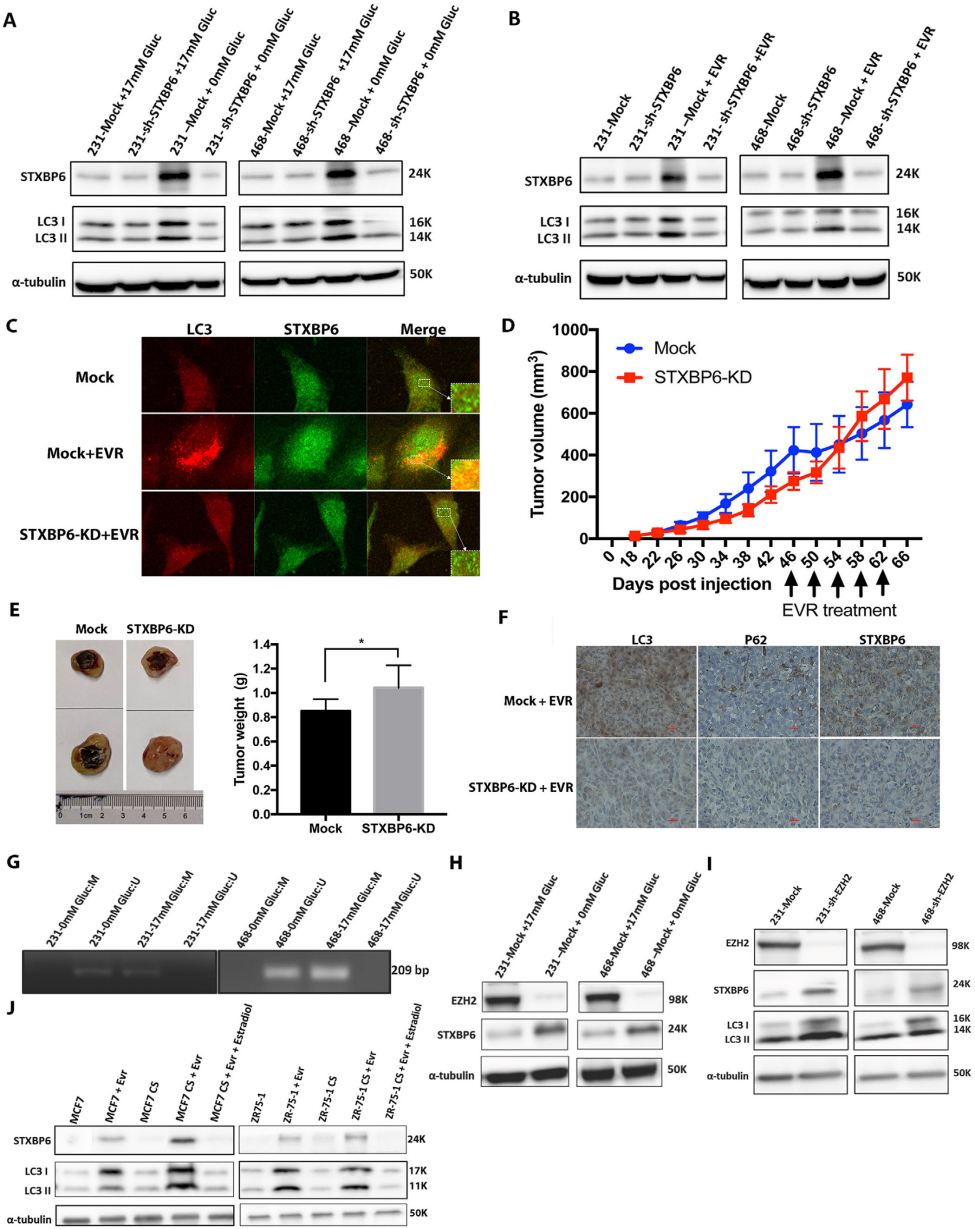
Autophagy induction rescues STXBP6 expression by inhibiting EZH2. Western blot analysis of LC3 and *STXBP6* expression in shRNA mediated abrogation of *STXBP6* (shSTXBP6) cells or mock cells cultured in media (A) with or without glucose or (B) with or without everolimus (EVR). Equal loading was assessed by a‐tubulin. C, Immunofluorescence analysis of endogenous LC3 and STXBP6 in MDA‐MB‐231 cells, everolimus‐treated MDA‐MB‐231 cells, and everolimus‐treated STXBP6‐silenced MDA‐MB‐231 cells. Cells were stained with rabbit anti‐LC3 and mouse anti‐STXBP6 antibodies, followed by anti‐mouse IgG‐AlexaFluor‐488 (green) and anti‐rabbit IgG‐AlexaFluor‐594 (red) secondary antibodies. Representative confocal images are shown at 40× magnification. D, Orthotopic tumor growth curve of *STXBP6* knockdown (STXPB6‐KD) and control (Mock) MDA‐MB‐231 cells after palpable tumor appeared. 1 mg/kg everolimus was injected intratumorally at 4‐day intervals from day 46 to day 62. E, Left: representative images of tumor size. Right: average tumor weight of each group. The tumor weight between Mock and STXBP6‐KD groups was compared by a *t*‐test. ^*^ *P *< .05. F, Immunohistochemical staining of STXBP6 and autophagy markers, LC3 and P62, in TNBC xenograft mice tumors after everolimus treatment. Magnification: 400×. G, Representative PCR product gel electrophoresis showing unmethylated *STXBP6* when TNBC cells are cultured without glucose compared with the cells cultured in normal conditions with 17 mM glucose. Primer sets used for amplification of *STXBP6* are designated as unmethylated (U) and methylated (M). H, Western blot analysis using anti EZH2 and STXBP6 antibodies reflecting the rescue effects of autophagy induction (glucose starvation) on STXBP6 through *EZH2* repression in MDA‐MB‐231 and MDA‐MB‐468 cells. Equal loading was assessed by a‐tubulin. I, Western blot analysis of LC3 and STXBP6 in MDA‐MB‐231 and MDA‐MB‐468 cells upon shRNA‐mediated abrogation of EZH2 (shEZH2). Equal loading was assessed by a‐tubulin. J, Western blot analysis of LC3 and STXBP6 in luminal breast cancer cells, cultured in normal media or in phenol‐red free media with charcoal stripped (CS) FBS under different condition: none treatment, everolimus or everolimus plus estradiol. Equal loading was assessed by a‐tubulin. Note that everolimus along with CS media are potential inhibitors of estrogen receptor. Increased levels of autophagy (LC3 levels) and *STXBP6* expression were observed upon everolimus treatment when they are cultured in CS media. Error bars represent the standard deviation of six replicates

The STXBP6 induction of autophagy in TNBC cells prompted us to examine whether STXBP6 is required for autophagy activation. In order to study the role of STXBP6 in autophagy, we used either glucose‐starved or everolimus‐added (mTOR inhibitor) STXBP6 stable knockdown cells to induce autophagy. The LC3‐II levels indicated that autophagy was not induced in STXBP6‐silenced cells in both conditions (Figures 6A,B). This finding was further verified by the distribution of the LC3 proteins using immunofluorescence (Figure 6C). In the TNBC mouse model, we injected everolimus into tumors generated from STXBP6 knockdown cells and mock cells, respectively, every 4 days. Mock tumors grew very slowly after everolimus treatment, whereas *STXBP6* knockdown tumors continued to grow (Figure 6D,E). IHC analysis of everolimus‐treated tumors showed that autophagy marker LC3 and STXBP6 were induced in mock tumors but not in *STXBP6* knockdown tumors (Figure 6F). Collectively, STXBP6 was not only essential for autophagy induction, but also upregulated by autophagy induction.

Intriguingly, when autophagy was activated under starvation and mTOR inhibition, *STXBP6* expression was also induced at RNA levels (Figure S11). As shown in Figure 2, promoter methylation controlled *STXBP6* expression. Thus, we performed methylation‐specific PCR to delineate the role of methylation behind the elevated *STXBP6* expression in autophagy condition. As shown in Figure 6G, we found decreased methylation levels under starved conditions in TNBC cells, whereas hypermethylation of the *STXBP6* promoter region was found in TNBC cells grown under normal conditions. These results indicate that demethylation of STXBP6 upon glucose starvation is the definitive cause behind increased *STXBP6* expression in autophagy condition.

As shown in Figure S2, H3K27ac is enriched at the promoter region of *STXBP6*. H3K27ac marked region is frequently occupied by EZH2 in tumor cells,^45^ which is significantly upregulated in breast cancer cells and promotes the transformation of breast cells.^46^ Further, it was reported that loss of EZH2 inhibited mTOR and induced autophagy.^47^ Therefore, we hypothesized that EZH2 silenced *STXBP6* through the hypermethylation of *STXBP6* promoter in TNBC. We first checked the dataset of EZH2‐binding genes^48^ and found that *STXBP6* is its bona fide target. We next examined EZH2 expression in glucose starved TNBC cells, and we found *EZH2* expression was inhibited while *STXBP6* expression was upregulated (Figure 6H). We knocked down *EZH2* expression in TNBC cells by shRNA against *EZH2*, and we found that *EZH2* silencing resulted in increased STXBP6 and autophagy activity (Figure 6I).

### 3.7 Altered autophagy and *STXBP6* expression via abrogation of estrogen receptor activity

To gain further molecular insights into STXBP6's specific role on autophagy induction in TNBC cells, we abrogated the estrogen receptor (ER) activity in luminal type of breast cancer cells, including MCF7 and ZR‐75‐1, by growing cells in estrogen‐free media (phenol‐red free media with charcoal‐stripped (CS) FBS). High levels of autophagy and STXBP6 upon everolimus treatment were found in luminal cells when environmental estrogen was removed (Figure 6J). However, the addition of estradiol antagonized the effect of everolimus and autophagy was repressed (Figure 6J). These results highlight the importance of the relationship between estrogen signaling and STXBP6‐mediated autophagy.

### 3.8 Inhibition of TNBC metastasis by STXBP6

**FIGURE 7:**
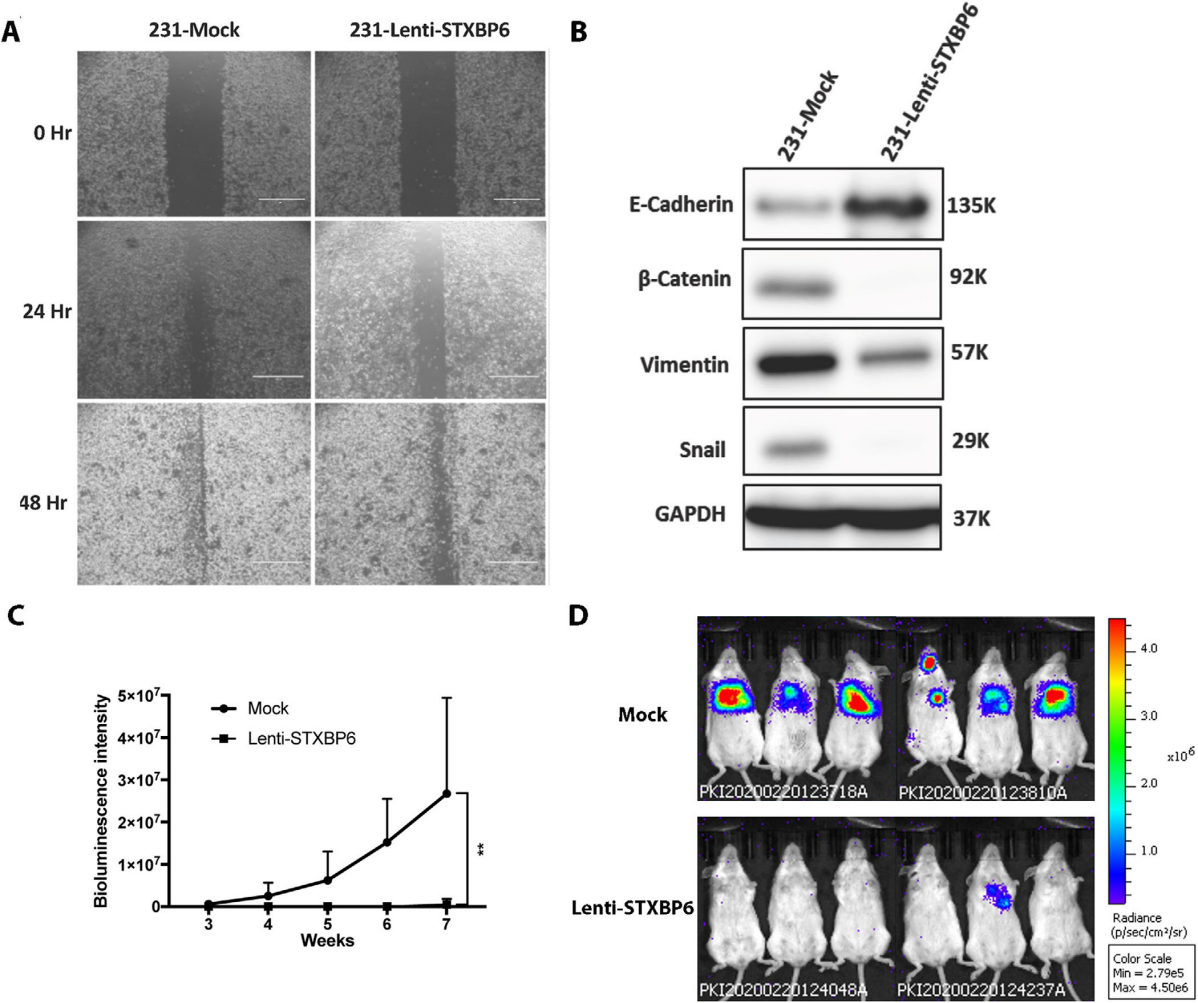
STXBP6 decreases the metastasis. A, Wound healing assay for migration analysis. Cell migration was analyzed by wound‐healing assay in MDA‐MB‐231 cells. STXBP6‐overexpressionover (Lenti‐STXPB6) compared with the control (Mock) and the cell migration distances were measured at 0, 24, and 48 h. B, Western blot analysis of expression of various epithelial–mesenchymal transition (EMT) regulators (E‐cadherin, β‐catenin, Vimentin, and Snail) in STXBP6‐overexpression (Lenti‐STXPB6) and control (Mock) MDA‐MB‐231 cells. Equal loading was assessed by GAPDH antibodies. C, Mean fluorescence index (MFI) of metastatic lesions in mice (n = 6) of control (Mock) and STXBP6‐overexpression (Lenti‐STXPB6) groups. The difference between these two groups are analyzed by a two‐way ANOVA. ^**^ *P *< .01. Error bars represent the standard deviation of six replicates. D, The distribution and fluorescence intensity of the metastatic tumors at week 7

Next, we investigated the effect of STXBP6 on the metastatic ability of TNBC cells. Wound healing assays showed that the Lenti‐STXBP6 cells distinctly migrated more slowly compared with the control (mock) cells (Figure 7A), suggesting the suppressive potential of STXBP6 on TNBC metastasis. Metastatic tumor cells usually endure an epithelial‐to‐mesenchymal transition (EMT), a cellular program that allows epithelial cells to convert to motile mesenchymal cells and promotes tumor cells metastasis, and a reversing of EMT would inhibit the metastatic trend of cells. We thus investigated the effect of *STXBP6* overexpression on EMT by examining the expression of EMT markers. We analyzed both the transcription‐level and protein‐level changes of various EMT markers, and showed that *STXBP6*‐overexpression significantly increased the expression of E‐cadherin, which facilitates adhesion formation and reverses EMT, whereas β‐catenin, Vimentin, and Snail were significantly suppressed (Figure 7B; Figure S12). We labeled the Lenti‐STXBP6 and mock MDA‐MB‐231 cells with luciferase and employed the experimental metastasis colonization mouse model by injecting them via intracardiac route. *STXBP6* expression significantly inhibited the formation of metastatic lesions and only one mouse formed a metastatic lesion in the lung (Figure 7C,D).

## 4 DISCUSSION

Although autophagy primarily acts as a tumor suppressive mechanism by maintaining genomic integrity and inhibiting cell proliferation, the roles of autophagy are context‐dependent in cancer. Our study establishes that an autophagy‐inducing protein STXBP6 is a suppressor of tumor growth and metastasis in TNBC only. STXBP6 and autophagy are mutually regulated in breast cancer cells: STXBP6 inhibits SNX27 function and triggers autophagy in the absence of ER activity, and autophagy activation induces *STXBP6* expression. Consistent with our findings that high *STXBP6* expression correlated with good clinical outcomes, ectopic expression of *STXBP6* in TNBC cells inhibited tumor growth and metastasis. Importantly, *STXBP6* expression was controlled by EZH2, which mediated its promoter methylation, thus providing multiple options to target autophagy as new treatment options for TNBC.

Currently, autophagy‐induced chemotherapy resistance in TNBC has been extensively studied. The role of autophagy in TNBC initiation and progression is not clear. Recent studies suggested that the deficiency of an essential autophagy gene was observed in TNBC only^49^ and a bacteria toxin‐activated autophagy existed in TNBC cells only.^50^ Both of these studies strongly suggested the existence of a TNBC‐specific mechanism of autophagy induction, which potentially inhibits TNBC cells only. Fully understanding this mechanism is the first step toward leveraging it as a strategic TNBC treatment. Here, we reveal a TNBC‐specific mutual regulation between STXBP6 and autophagy. Our study linked epigenetic regulation to a TNBC‐intrinsic autophagy process. Recent studies demonstrate that epigenetic modifications determined the expression of autophagy‐related genes and autophagy levels in both normal and cancer cells.^51^, ^52^ Epigenetic regulators include the methyltransferase, EZH2, which is recruited to gene promoters of several upstream mTOR inhibitors. EZH2 methylates and silences these autophagy‐activating promoters and its inhibition induces autophagy.^53^ Under basal conditions, the methyltransferase, G9a, interacts with autophagy proteins LC3B, WIPI1, and DOR gene promoters, and it represses gene expression.^54^ Moreover, inhibition of the HMT (Histone methyl transferase), EZH2 or G9a promotes autophagy in cancer cells while the use of a histone deacetylase inhibitor gave contradictory results in cancer cells.^52^, ^55^ In the present study, we demonstrated that knocking down EZH2 led to the rescue of *STXBP6* expression and further led to autophagy induction. Interestingly, EZH2 was inhibited and *STXBP6* methylation was impaired when cells were subjected to glucose starvation, leading to autophagy activation. These observations may help us to better understand how epigenetic changes repress autophagy. Previous studies also reported that starvation removes methylation marks on histones and activates autophagosome formation.^54^ Therefore, our findings may facilitate designing new treatments based on methylation‐controlled STXBP6‐mediated autophagy for TNBC.

The precise role of autophagy in TNBC promotion and metastasis remains controversial. It has been shown that the deficiency of autophagic components such as Beclin 1 and ATG4C promotes the development of malignancy.^21^, ^56^ We found that the silencing of the tumor‐suppressor gene *STXBP6* distinctly impaired the autophagic process in TNBC. Autophagy is a major biological process regulated by mTOR signaling. The positive correlation of *STXBP6* expression with the autophagy that we observed led us to investigate whether STXBP6 triggers autophagy in TNBC cells by inhibiting mTOR pathway. Accordingly, we explored downstream targets of the mTOR pathway in *STXBP6*‐overexpressed cells versus controls. Overexpression of STXBP6 significantly suppressed the phosphorylation of p70 S6 Kinase and 4E‐BP1, downstream targets of mTOR. Moreover, STXBP6 is highly possibly involved in extracellular stimuli‐induced autophagy, because starvation and pharmacological challenging of mTOR pathway not only induced *STXBP6* expression but also triggered autophagy. PI3K plays an important role in controlling mTOR activation, and 3‐MA inhibits autophagy by blocking autophagosome formation by targeting class III PI3K.^57^ Accordingly, we identified growth recovery of STXBP6‐overexpressed TNBC cells upon treatment with 3‐MA, which could be accused of the activation of mTOR. These findings emphasize that STXBP6 suppresses the growth rate of TNBC cells by inactivating the mTOR pathway, leading eventually to activation of autophagy. Rapamycin and its analogues are well‐characterized autophagy‐inducing drugs^58^ and currently are tested for cancer treatment.^59^ Similar to our findings, targeting CXCR4‐mTOR signaling by rapamycin resulted in autophagic cell death and decreased metastasis in gastric cancer models.^60^

Several signaling pathways regulate autophagy. We successfully revealed that SNX27, GRP78, and identified other endoplasmic reticulum (ER)‐stress‐associated proteins that interact with STXBP6. ER‐stress could trigger autophagy and induce the expression of its related genes. The effects of STXBP6 overexpression on cell proliferation, EMT, autophagy, and mTOR signaling were the same as the reports on *SNX27* knock‐out models,^41^, ^61^ suggesting that STXBP6 and SNX27 interact with each other antagonistically. In addition, *SNX27* was overexpressed in patients with invasive breast cancer and involved in facilitating metastasis.^42^ Targeting EMT processes is promising in developing anticancer drugs.^62^ Thus, we investigated STXBP6's role in mediating the alteration of EMT regulators in TNBC metastasis. Overexpression of *STXBP6* indeed reversed the expression of EMT markers and significantly reduced metastasis. These findings suggest that STXBP6 should be considered as a potential molecular target for controlling TNBC progression.

*STXBP6* failed to induce autophagy when it was overexpressed in luminal cells, and this prompted us to turn our attention to exploring estrogen receptor association in STXBP6‐mediated autophagy. To this end, we employed luminal cells and inactivated their ER activity by culturing them in estrogen free media. We found that abrogation of ER activity rescued pharmacological (everolimus) autophagy induction in luminal cells, which might be associated with the ability of everolimus to reduce ER transcriptional activity.^63^ These findings strongly indicate that ER activity in luminal cells probably repressed the ability of STXBP6 to induce autophagy.

In summary, our studies unveil a role of STXBP6 in TNBC that highlights a new paradigm in autophagy regulation. Enforced expression of an autophagy gene not only promotes autophagy in breast cancer cells but also inhibits their proliferative potential, which indicates that autophagy may be a fundamental mechanism for controlling deregulated tumor cells. Further studies should delineate the signal pathways that mediate the mutual regulation between autophagy and STXBP6 in tumorigenesis. Nonetheless, decreased expression of *STXBP6* in human breast carcinoma suggests that specific molecular alterations in autophagy pathways may contribute to tumorigenesis. The identification of this novel autophagy‐related protein provides important insights into mechanistic paradigms of aberrant autophagy in breast malignancies.

## AUTHOR CONTRIBUTIONS

GL, JS, and LC contributed to the study design and writing the manuscript; GL, JS, and SA performed the biochemical and cellular analyses; JS and MS designed the mouse model experiments; NG and FS performed the mass spectrometry analyses; SZ and KS performed the in silico methylation analysis; SB, IAB, and SG provided clinical specimen; NH, DB, and NAK performed survival analyses; AR and AD performed the docking analyses, GL, JS, NH, XM, AR, and LC contributed to the data analyses.

## CONFLICT OF INTEREST

The authors declare no potential conflicts of interest.

## Supporting information

Supporting Information

Supporting Information

Supporting Information

Supporting Information

## Data Availability

Authors agreed to share all the data reported in this article including protocols, methods and material.
