# Supplementary material for: STXBP6, reciprocally regulated with autophagy, reduces triple negative breast cancer aggressiveness
Source: Clin Transl Med. 2020 Aug 11;10(3):e147. doi: 10.1002/ctm2.147 (PMC7418817; doi:10.1002/ctm2.147)
Supplement: Supplementary file 1 — Supporting Information [file CTM2-10-e147-s001.pdf]

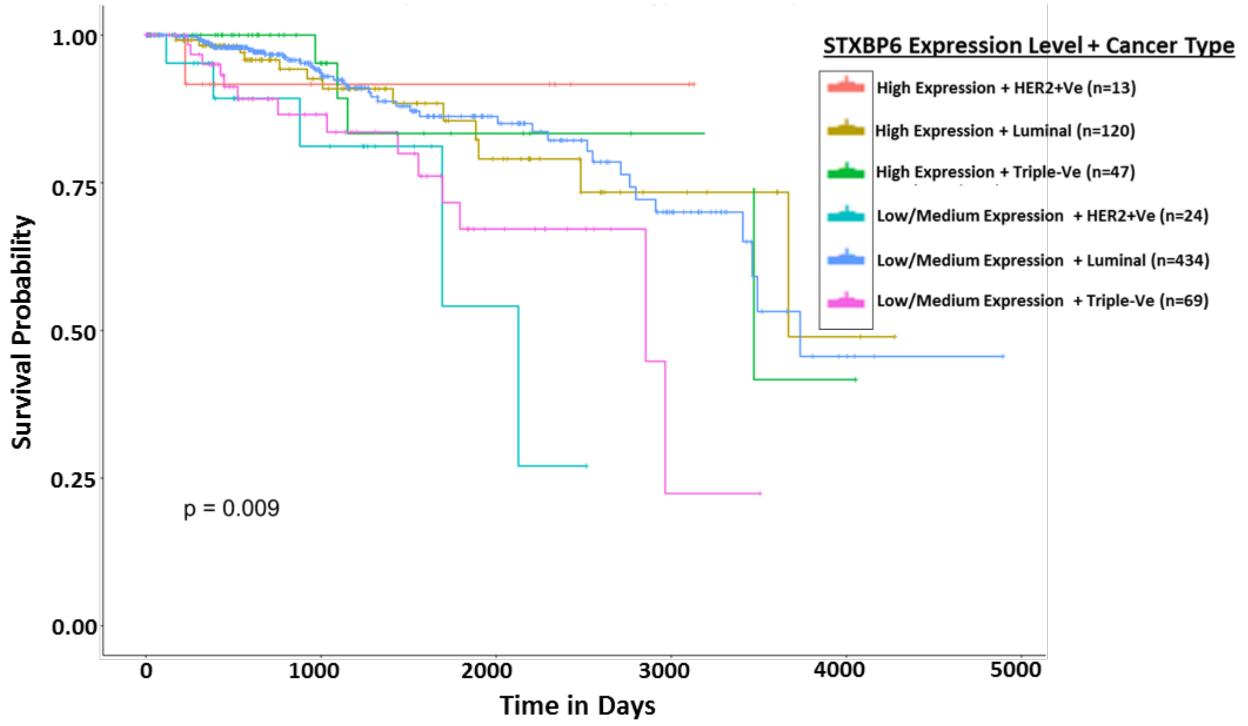

Figure S1. STXBP6 gene expression is associated with overall survival in different subtypes of breast cancer. Survival risk curves are shown for STXBP6 expression in breast cancers using TCGA cohort. In TCGA, survival curves are drawn for different subtypes of (HER2^{+}Ve, Luminal and Triple negative breast cancer patients based on STXBP6 expression levels (high vs. low, using the median as cutoff) expression levels.

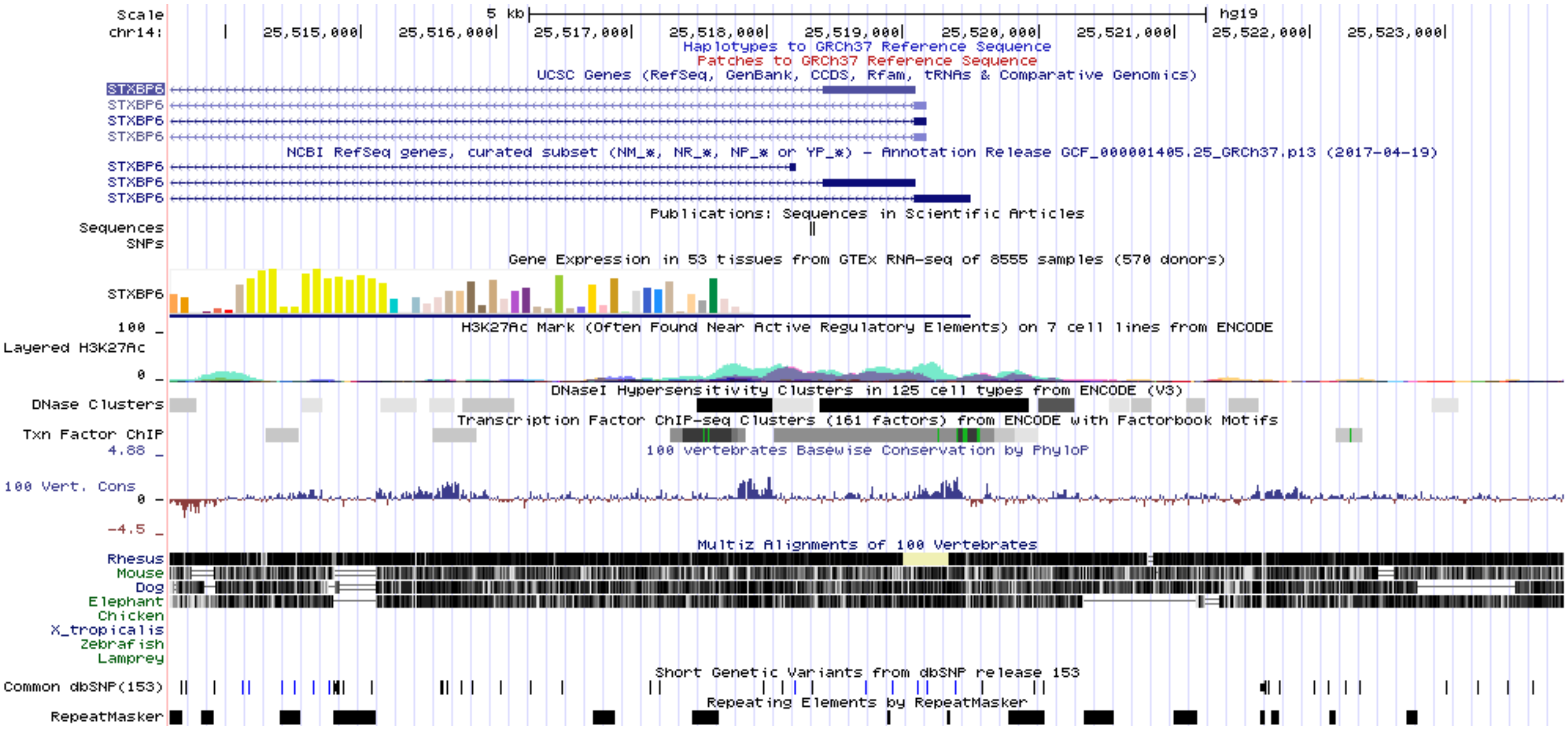

Figure S2. UCSC genome browser snapshot of STXBP6 promoter region

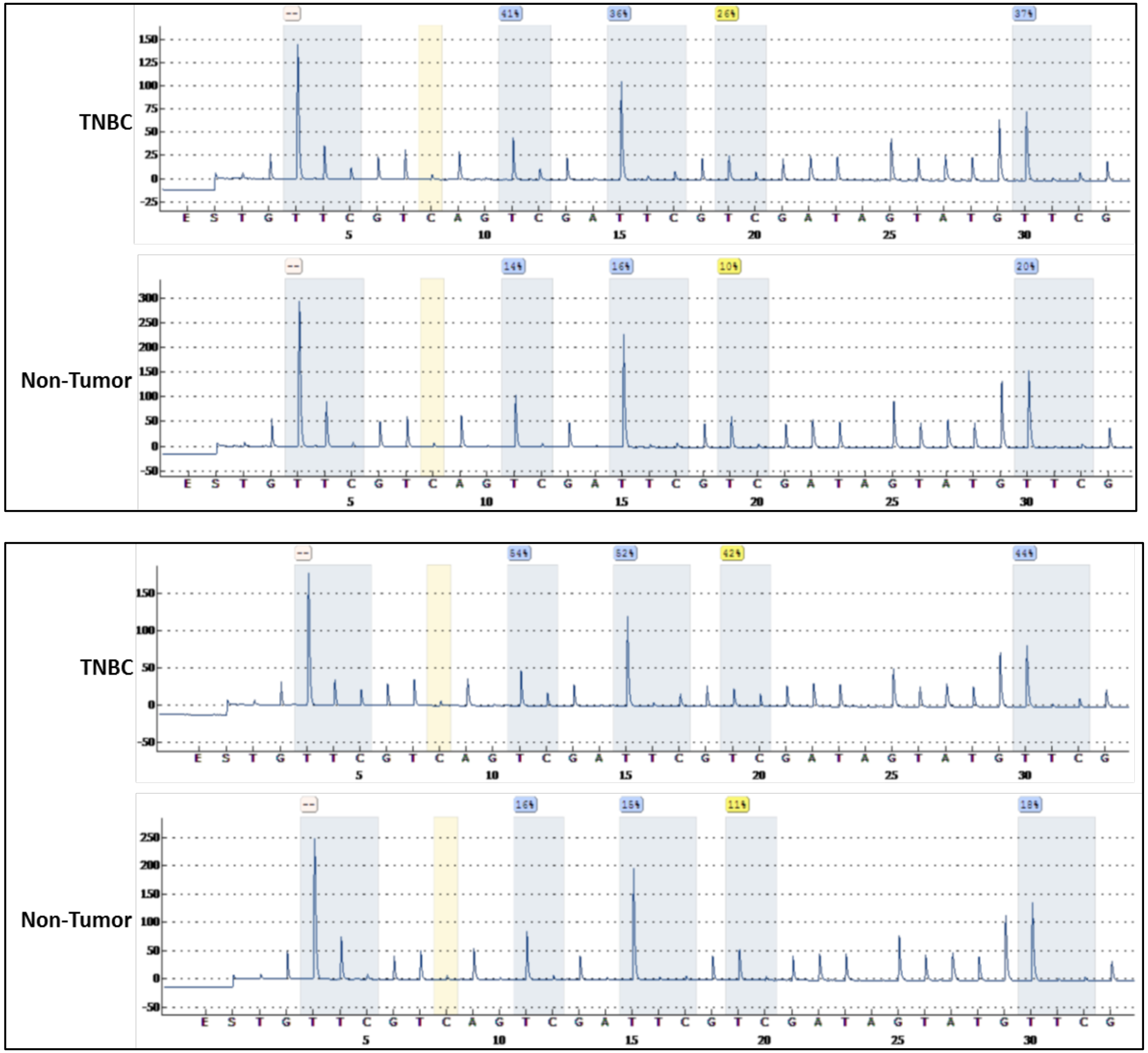

Figure S3. Pyrograms representing the methylation difference of CpG sites in the 5'-UTR of STXBP6 between TNBC tissue and the corresponding non-malignant breast tissue (Non-Tumor). Pyrograms for two pairs of specimen are shown as examples.

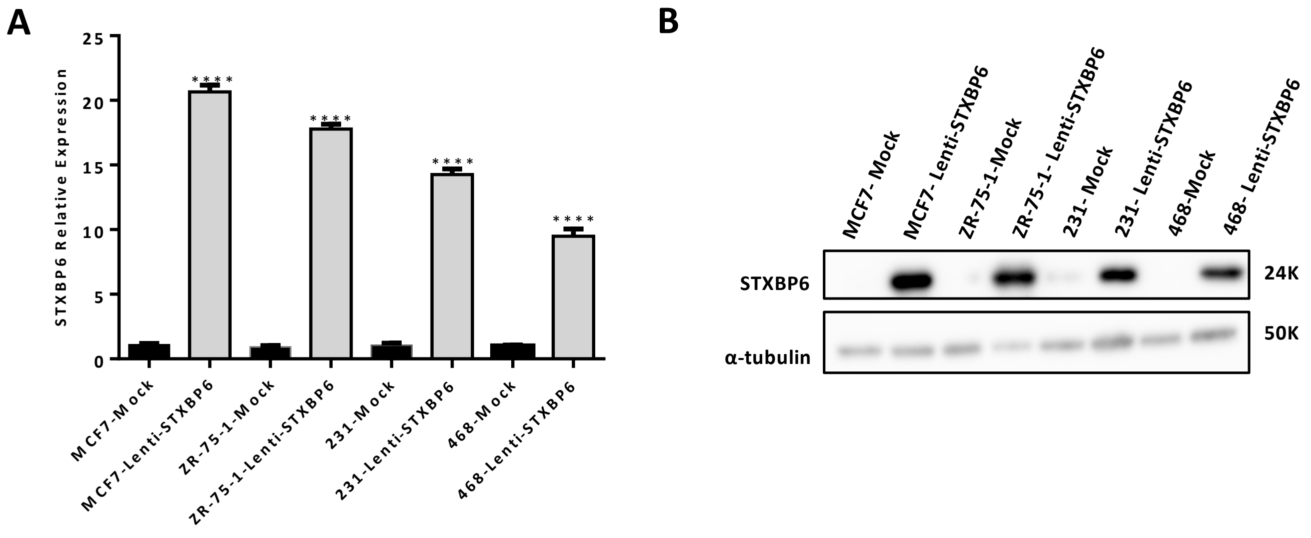

Figure S4. Confirmation of STXBP6 ectopic expression in breast cancer cells infected with lenti-STXBP6 (a lentivirus vector carrying the *STXBP6* gene) or with its corresponding mock. (A) STXBP6 mRNA level ectopic expression in MCF7 and ZR-75-1 luminal cells and MDA-MB-231 and MDA-MB-468 TNBC cells. Relative STXBP6 expression was calculated by $2^{-\Delta Ct}$. **** P<0.0001. **(B)** Western blotting on cell lysate of luminal and TNBC cells. Equal loading was assessed by α-tubulin antibody.

A

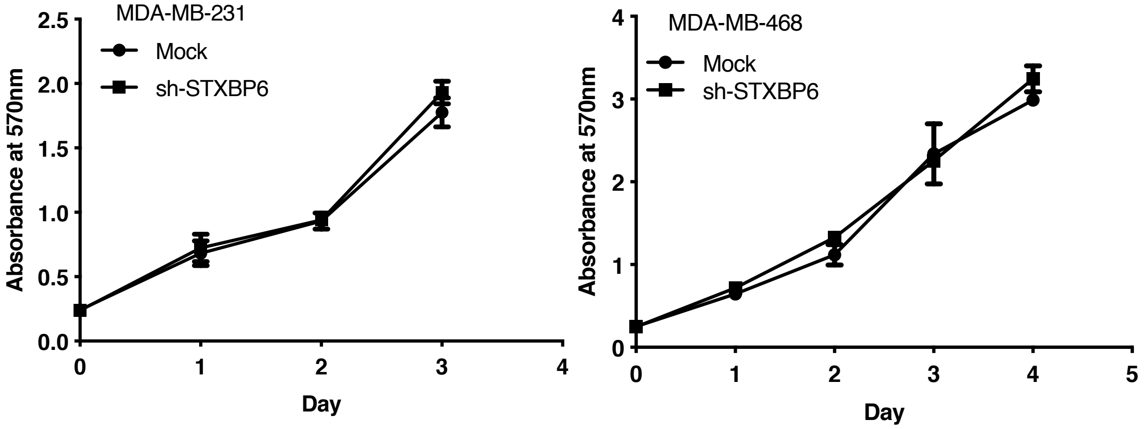

B

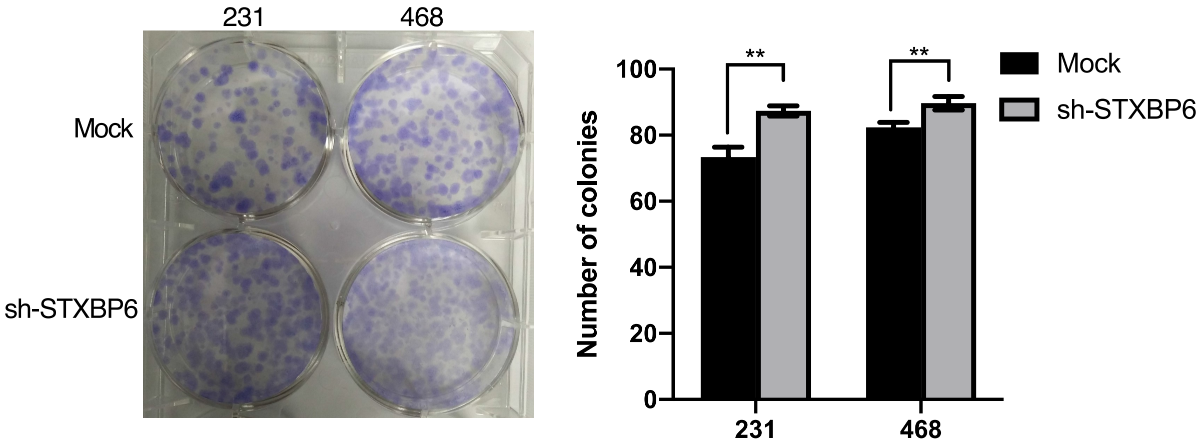

Figure S5. Effect of shRNA mediated knockdown of STXBP6 on cell proliferation and clonogenic survival. (A) Proliferation assays of STXBP6 knockdown (sh-STXPB6) TNBC cells versus empty vector (Mock). Cells at a concentration of 5,000 cells per well were seeded in the 96-well plate and incubated for indicated time. The quantity of viable cells was determined by MTT assay. The difference between mock group and sh-STXBP6 group was calculated by a two-way ANOVA. (B) Colony formation assays of MDA-MB-231 and MDA-MB-468 TNBC cells of STXBP6 knockdown (sh-STXPB6) compared to the control (Mock). Left, the whole-well images of clonogenic cells; right panels, the average number of colonies. Error bars represent the standard deviation of biological triplicates.The difference between mock group and sh-STXBP6 group was analyzed by a t-test. ***P*<0.01.

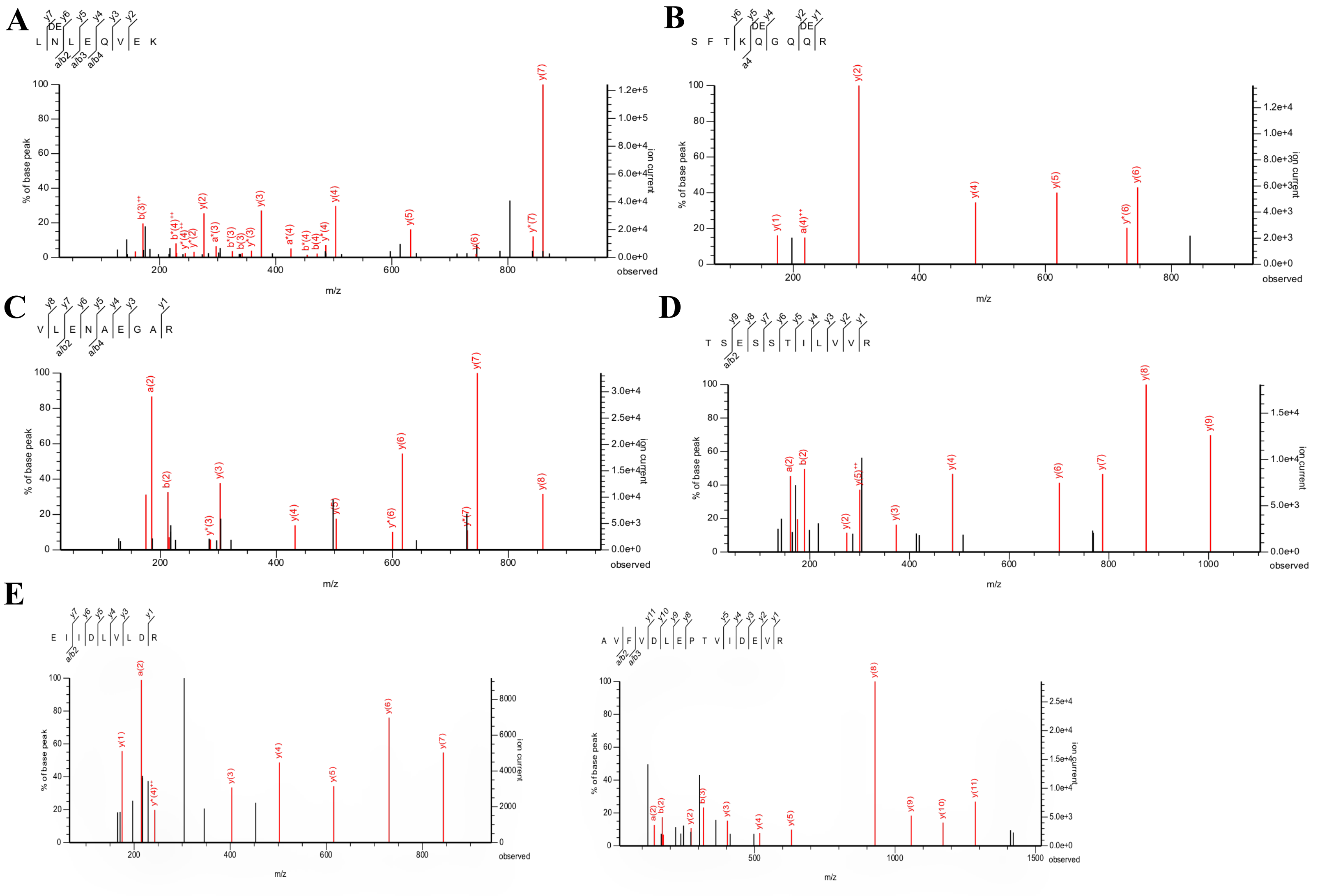

Figure S6 . Data-dependent MS/MS sequencing scan of top hits of STXBP6 interacting peptides: (A) ABCAD, (B) BCE1, (C) GRP75, (D) SPNXA, (E) TBA1A.

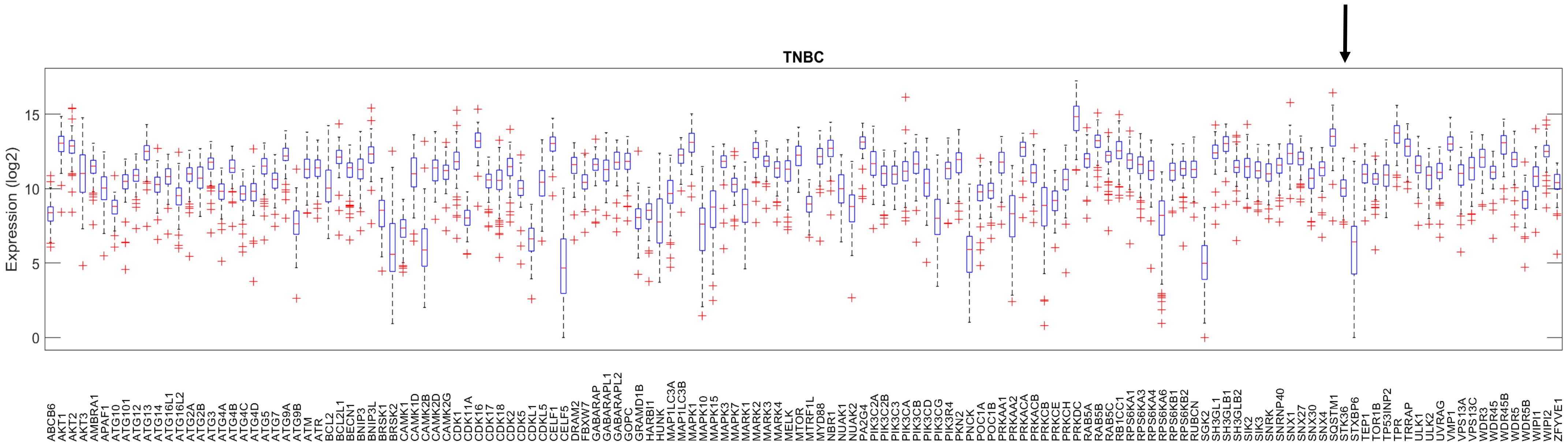

Figure S7. Expression of Autophagy Genes in TNBC. The arrow points to STXBP6. Note that STXBP6 expression is among the lowest expressed genes autophagy gene. The box central red mark indicates the median and the box bottom and top edges indicate the 25th and 75th percentiles. The whiskers (dotted lines) extend to the most extreme data points not considered outliers, and the outliers are plotted individually using the '+' symbol. The outliers correspond to deviations from the mean of more than +/–2.7 standard deviations assuming normally distributed data.

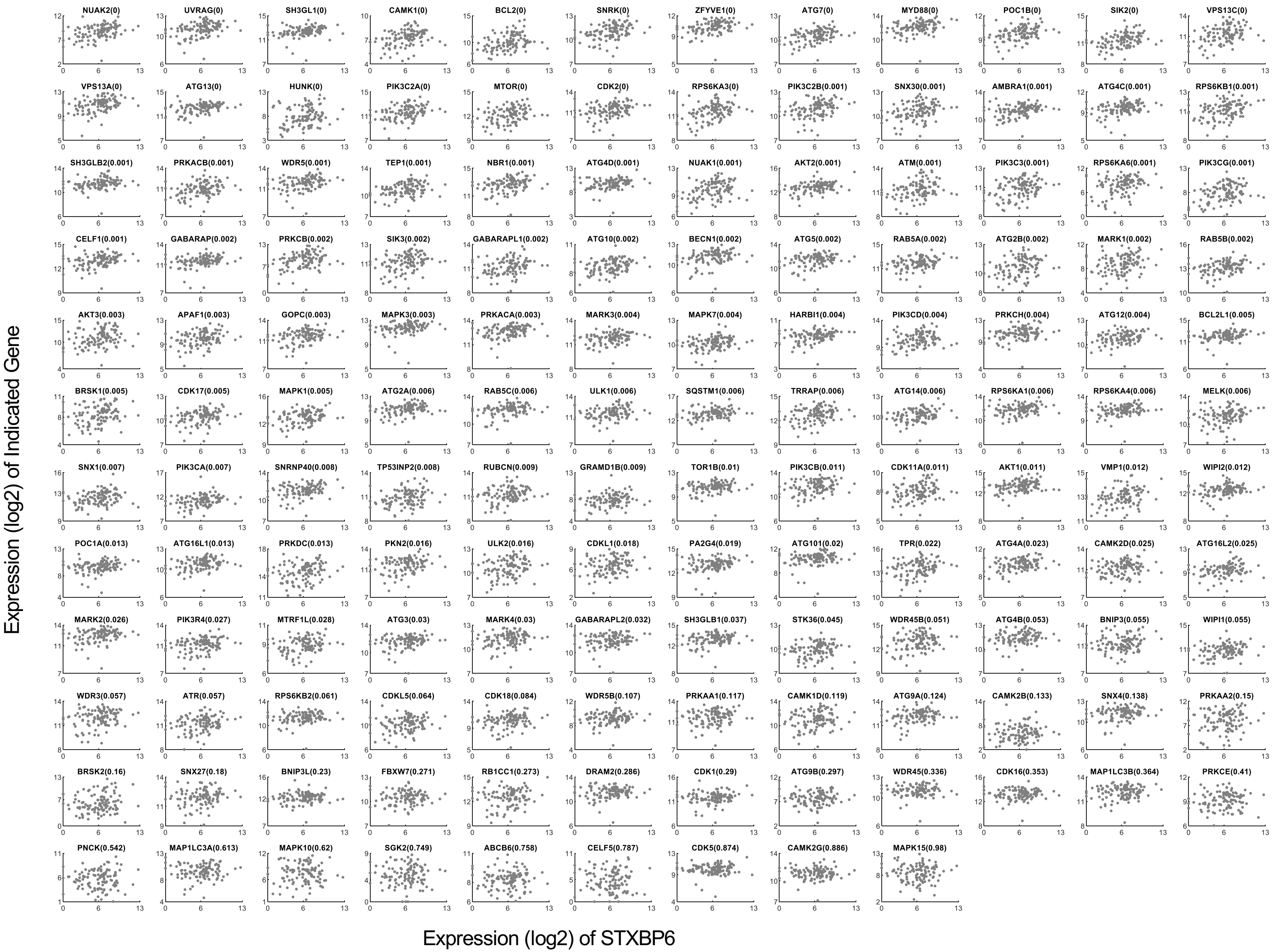

Figure S8. Correlations of All Autophagy Genes to STXBP6 (Kendall Tau Correlation of Log2-scale, sorted by P-value). The title shows the gene being correlated to STXBP6 and the p-value of the correlation.

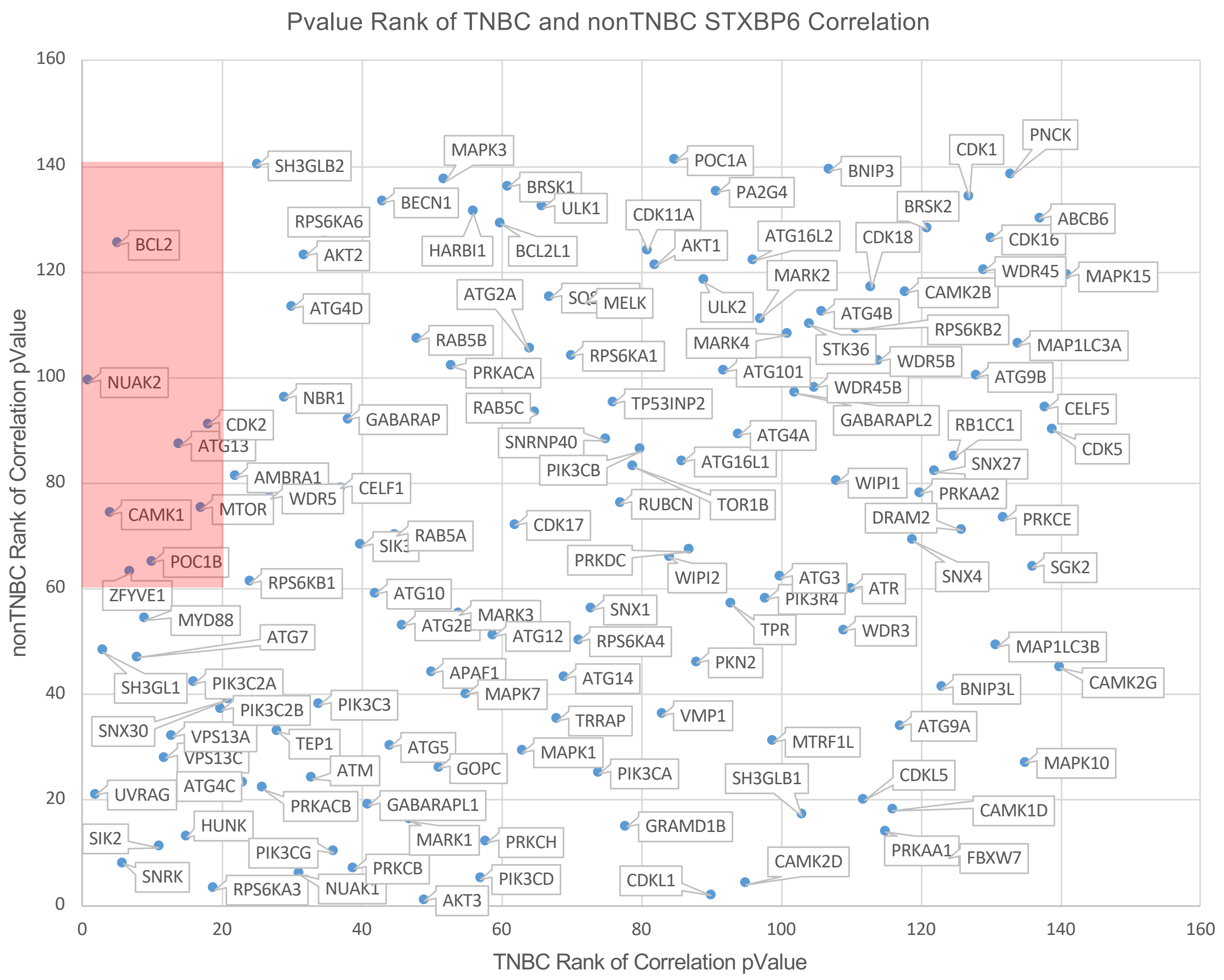

Figure S9. Comparison of STXBP6 Correlations between TNBC and Non-TNBC. Red box highlights the genes most varying with STXBP6 only in TNBC.

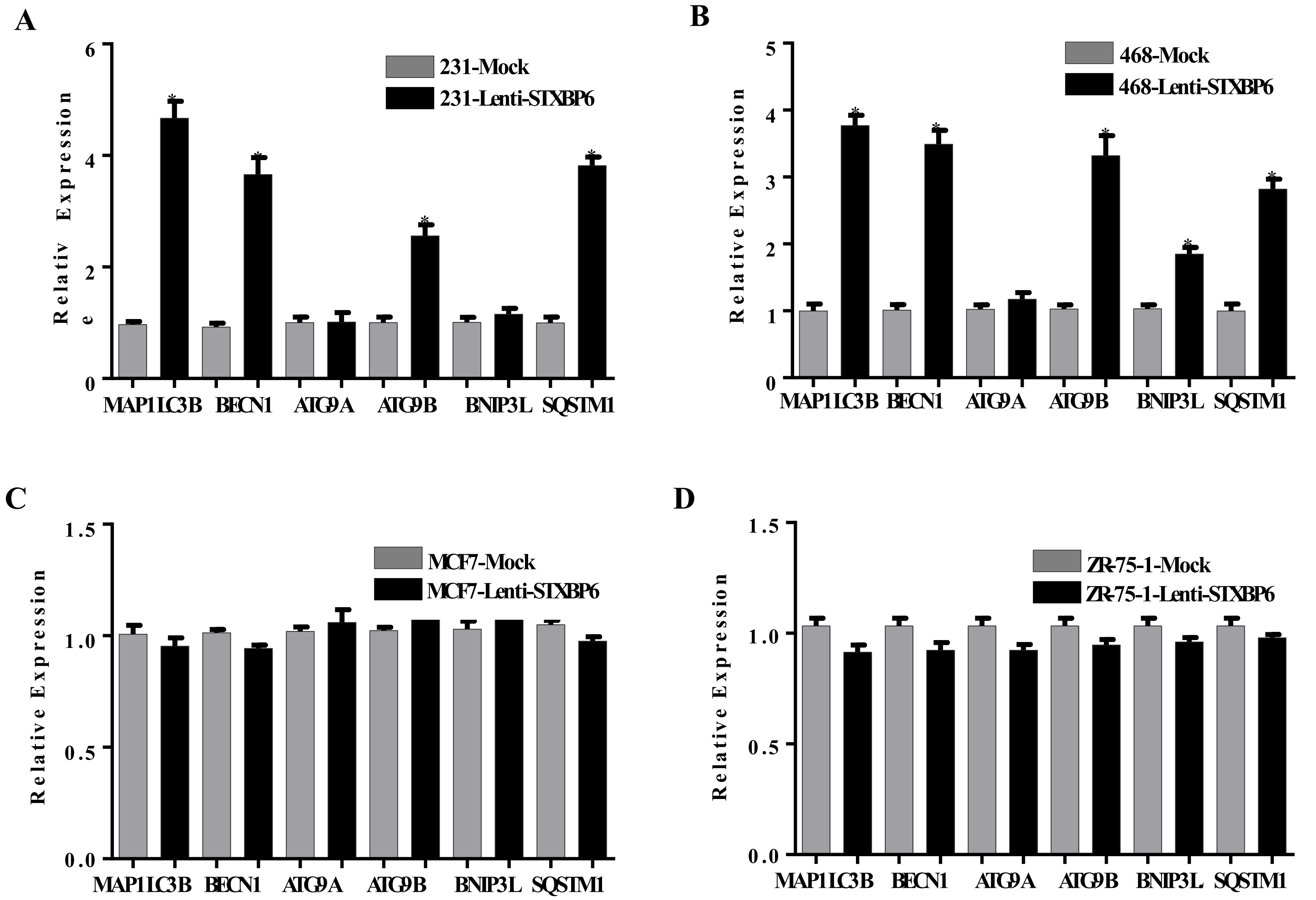

Figure S10. qPCR analysis revealed upregulation of autophagy related genes in TNBC cells with ectopic STXBP6-mediated overexpression of STXBP6 (Lenti-STXPB6). (A) and MDA MB-468 (B) TNBC cells and in MCF7 (C) and ZR-75-1 (D) luminal subtype breast cancer cells. HPRT1 was used as an internal control. Relative mRNA expression of MAP1LC3B (LC3B), BECN1, ATG9A, ATG9B, BNIP3L and SQSTM1(SQST) were compared between Lenti-STXPB6 and control (Mock) groups.

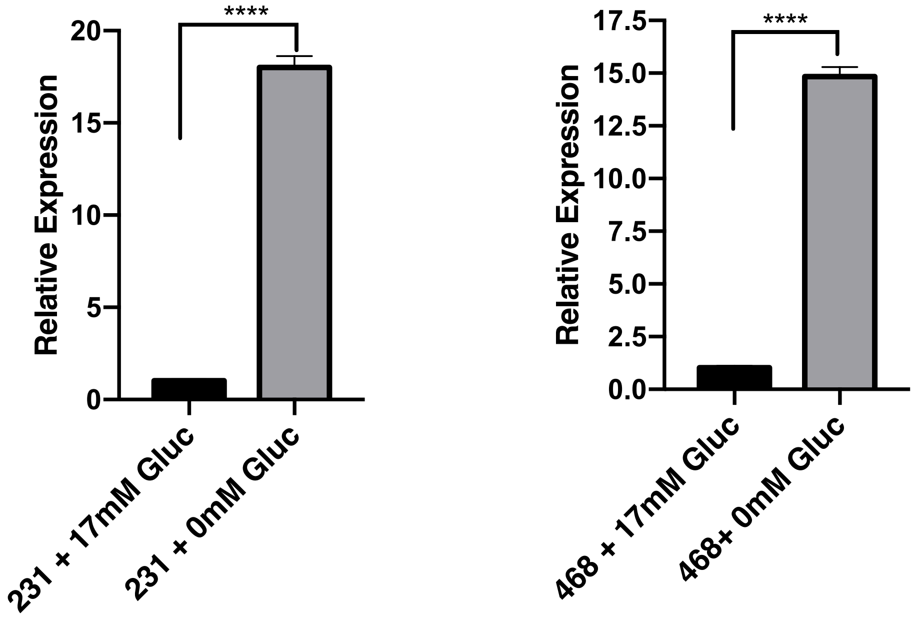

Figure S11. Starvation induced the expression of STXBP6 mRNA in TNBC cells. MDA-MB-231 and MDA-MB-468 cells were cultured in growth media with or without glucose for 48 hours. RNA were extracted and qRT-PCR were used to quantify STXBP6 expression. ****, $P<0.0001$.

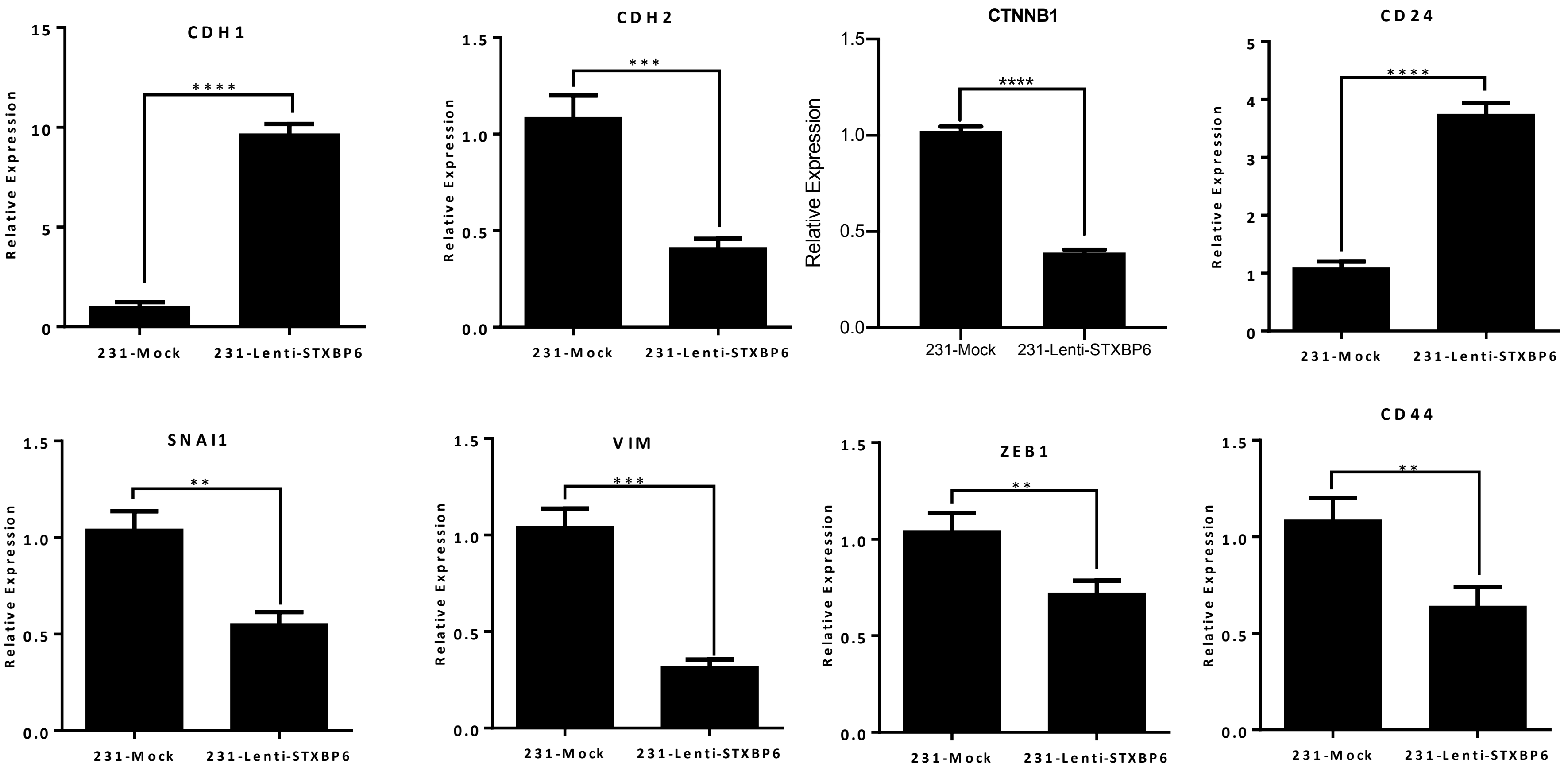

Figure S12. qPCR analysis of different EMT regulators in ectopic STXBP6-mediated overexpression of STXBP6 (Lenti-STXPB6) and control (Mock) MDA-MB-231 cells. HPRT1 was used as an internal control. Relative expression levels of all genes in STXBP6 overexpressed cells were normalized against mock cells. The EMT regulator genes including CDH1, CDH2, CTNNB1, CD24, SNAI1, VIM, ZEB1 and CD44 were analyzed. Bars represent the means ± SDs of biological triplicates. The difference between two groups was analyzed by a t-test. ***P*<0.01, ****P*<0.001 and **** *P*<0.0001.
