# Supplementary material for: STXBP6, reciprocally regulated with autophagy, reduces triple negative breast cancer aggressiveness
Source: Clin Transl Med. 2020 Aug 11;10(3):e147. doi: 10.1002/ctm2.147 (PMC7418817; doi:10.1002/ctm2.147)
Supplement: Supplementary file 4 — Supporting Information [file CTM2-10-e147-s003.docx]

**Table S3.** **Primers for qRT-PCR analysis**

| **Gene Symbol** | **Gene Description** | **Primers (Forward/Reverse)** |
| --- | --- | --- |
| *ATG9A* | Autophagy related 9A | 5’-TTTGCGTTAGGGTGAAGACC-3’ |
|  |  | 5’-AGGGCAGCAAAGTATTTCCA-3’ |
| *ATG9B* | Autophagy related 9B | 5’-CCTTGGGCAGTTCTTCTTTG-3’ |
|  |  | 5’-CTTCCTGGTGCCTGGTACAT-3’ |
| *BECN1* | Beclin 1 | 5’-TCACCATCCAGGAACTCACA-3’ |
|  |  | 5’-CCTGGCGAGGAGTTTCAATA-3’ |
| *MAP1LC3B (LC3B)* | Microtubule associated protein 1 light chain 3 beta | 5’-CGGAAAGCAGCAGTGTACCA-3’ |
|  |  | 5’-GGCAGAAGGGAGTGTGTCTGA-3’ |
| *BNIP3L (NIX)* | BCL2 interacting protein 3 like | 5’-AAGGCAGGCTTCATTTTTCA-3’ |
|  |  | 5’-CCAATAATTTCCACAACGGG-3’ |
| *SQSTM1* | Sequestosome 1 | 5’-ATCGGAGGATCCGAGTGT-3’ |
|  |  | 5’-TGGCTGTGAGCTGCTCTT-3’ |
| *STXBP6* | Syntaxin binding protein 6 (amisyn) | 5'-GCCAGCACAGCGTCAGAA-3' |
|  |  | 5'-TGGCAGGTATGGTGGAGGAT-3' |
| *CDH1* | Cadherin 1, E-cadherin | 5'-AAGAAGGAGGCGGAGAAGAG-3' |
|  |  | 5'-GGCTGTGGGGTCAGTATCAG-3' |
| *CDH2* | Cadherin 2, N-cadherin | 5'-ATCCGACGAATGGATGAAAG-3' |
|  |  | 5'-CATAGTCCTGCTCACCACCA-3' |
| *CTNNB1* | Catenin beta 1 | 5’- GACCTCATGGATGGGCTG-3’ |
|  |  | 5’- AGGCTAGGGTTTGATA-3’ |
| *CD24* | CD24 molecule | 5'-TGCTCCTACCCACGCAGATT-3' |
|  |  | 5'-GGCCAACCCAGAGTTGGAA-3' |
| *SNAI1* | Snail | 5'-CTTCCAGCAGCCCTACGAC-3' |
|  |  | 5'-CGGTGGGGTTGAGGATCT-3' |
| *VIM* | Vimentin | 5'-GAGAACTTTGCCGTTGAAGC-3' |
|  |  | 5'-CCAGAGGGAGTGAATCCAGA-3' |
| *ZEB1* | Zinc finger E-box binding homeobox 1 | 5'-GCACCTGAAGAGGACCAGAG-3' |
|  |  | 5'-TGCATCTGGTGTTCCATTTT-3' |
| *CD44* | CD44 molecule | 5'-GCATTGCAGTCAACAGTCGAAGAAG-3' |
|  |  | 5'-GGCCTCTCCGTTGAGTCCACTT-3' |
| *HPRT1* | Hypoxanthine Phosphoribosyltransferase 1 | 5’-TGACACTGGCAAAACAATGCA-3’ |
|  |  | 5’-GGTCCTTTTCACCAGCAAGCT-3’ |
